# Pathophysiology, conventional treatments, and evidence-based herbal remedies of hair loss with a systematic review of controlled clinical trials

**DOI:** 10.1007/s00210-025-04286-6

**Published:** 2025-06-19

**Authors:** Anood T. Allam, Riham A. El-Shiekh, Ahmed M. El-Dessouki, Noha M. Gamil, Nehal Mohamed Eisa, Menna M. Ayoub, Weam A. M. Khallil, Mariam A. N. Farag, Madonna G. Attallah, Mohamed S. Abd E. L. Hafeez, Dina Abou-Hussein

**Affiliations:** 1 Clinical Pharmacy Department, Faculty of Pharmacy, Cairo University, Cairo, Egypt; 2 Department of Pharmacognosy, Faculty of Pharmacy, Cairo University, Cairo, 11562 Egypt; 3 Pharmacology and Toxicology Department, Faculty of Pharmacy, Ahram Canadian University, 6 Th of October City, Giza, 12566 Egypt; 4 Department of Pharmacology and Toxicology, College of Pharmaceutical Sciences and Drug Manufacturing, Misr University for Science and Technology (MUST), P.O Box 77, Giza, Egypt; 5 Clinical research department at Giza Health Affairs Directorate, MOHP, Giza, Egypt; 6 Department of Pharmacognosy, Faculty of Pharmacy, Egyptian Russian University, Cairo-Suez Road, Badr City, , 11829 Cairo Egypt; 7 Biochemistry Department, Faculty of Pharmacy, Badr University in Assiut, Assiut, Egypt; 8 Clinical Pharmacy Department, Faculty of Pharmacy, Badr University in Assiut, Assiut, Egypt; 9 Biochemistry Department, Faculty of Pharmacy, Egyptian Russian University in Cairo, Cairo, Egypt; 10 Department of Pharmacy, Kut University College, Al Kut, Wasit, 52001 Iraq

**Keywords:** Hair loss, Herbal remedies, Pathophysiology, Genetic factors, Systematic review

## Abstract

Hair loss is a distressing problem that affects the self-image and quality of life of many people. The increasing demand for alternative treatments has led to growing interest in using herbal medicine for hair loss management. This literature review is divided into two main parts: a narrative overview and a systematic synthesis, evaluating the efficacy of herbal remedies, gathering findings from recent clinical studies, and traditional practices. The narrative section explores the pathophysiology of hair loss, contributing factors, and conventional pharmacological treatments, including their limitations and adverse effects. The second part presents a systematic review of sixteen randomized controlled trials evaluating the clinical efficacy and safety of herbal remedies for hair loss. Natural products such as rosemary, green tea, ginseng, *Aloe vera*, olive, and saw palmetto have shown promising efficacy in promoting hair growth, improving hair density, reducing hair shedding, and enhancing patient satisfaction through mechanisms involving anti-inflammatory, hormonal, and enhanced scalp circulation. However, study design limitations and outcome variability necessitate further rigorous, standardized research. This review aims to offer an integrative, evidence-informed perspective on the therapeutic potential of herbal medicine for hair loss in clinical evidence.

**Supplementary Information:**

The online version contains supplementary material available at 10.1007/s00210-025-04286-6.

## Introduction

Hair loss, clinically referred to as alopecia, affects millions worldwide, manifesting in various forms such as androgenetic alopecia (AGA), alopecia areata (AA), and telogen effluvium (TE). Although there are effective pharmacologic therapies, such as minoxidil and finasteride, there is still a better way to handle hair loss while mitigating their multiple side effects and low efficacy among some patients (Phillips et al. 2017). Nutritional deficiency is one of the leading causes of alopecia. Vitamins (A, B-complex, C, D, and E), minerals (zinc, iron, selenium, and copper), and essential fatty acids have key functions in preserving the functionality of the hair follicles (Shapiro et al. 2000). Protein, the building block of hair, is also critical in hair structure and strength. Lack of these nutrients can impact the structural integrity of the hair shaft, the follicles’ strength, and hair growth rate (Mubki et al. 2014). For instance, iron deficiency is a known precipitating factor for TE, especially in women, and vitamin D deficiency has also been associated with autoimmune-mediated alopecia, i.e., AA (Camacho-Martinez 2009). In addition, lifestyle variables, like stress, nutrition, and scalp hygiene, contribute to hair wellness. Chronic stress is a major trigger for hair-shedding conditions such as TE, as elevated cortisol levels can disrupt the hair growth cycle and lead to premature follicular regression (Mounsey and Reed 2009). Furthermore, subpar scalp treatments, excessive applications of chemical treatments, and tight hairstyles can aggravate hair loss by damaging hair follicles and constricting scalp blood flow. Habits, mainly smoking, alcohol consumption, and irregular sleep, can also have a similar effect in causing a compromised hair structure and slower hair growth (Strazzulla et al. 2018).

Apart from the current medical advances, historical and traditional methods of hair loss management have an important role in various communities (Lourith and Kanlayavattanakul 2013). Traditional herbal medications, e.g., black seed oil, rosemary oil, and Bhringraj, were used to promote growth, scalp health, and hair strength. Ayurvedic, Chinese, Middle Eastern, African, and Native American practices are abundant with natural remedies, which are increasingly supported by contemporary scientific evidence. For example, rosemary oil has been reported to be as effective as minoxidil in stimulating hair regrowth, etc., on the other hand, black seed oil, due to its antimicrobial activity, is beneficial for scalp conditions (Gasmi et al. 2023).

Herbal preparations have always been fundamental to traditional systems of medicine around the globe, and many herbs are claimed to promote hair growth and arrest hair loss (Zgonc Škulj et al. 2020). Herbs contain compounds presumed to exert therapeutic effects, including scalp health enhancement, hormonal modulation, and oxidative stress reduction. Despite extensive uses, scientific evidence for these assertions is mixed (Shen et al. 2018). This review seeks to critically assess the efficacy of herbal medicines in hair loss treatment, exploring their mechanisms of action and summarizing evidence from recent studies. It also contributes to a better understanding of herbal medicines’ potential role in hair loss management by identifying the strengths and limitations of the existing research.

### Hair loss etiologies

**Fig. 1:**
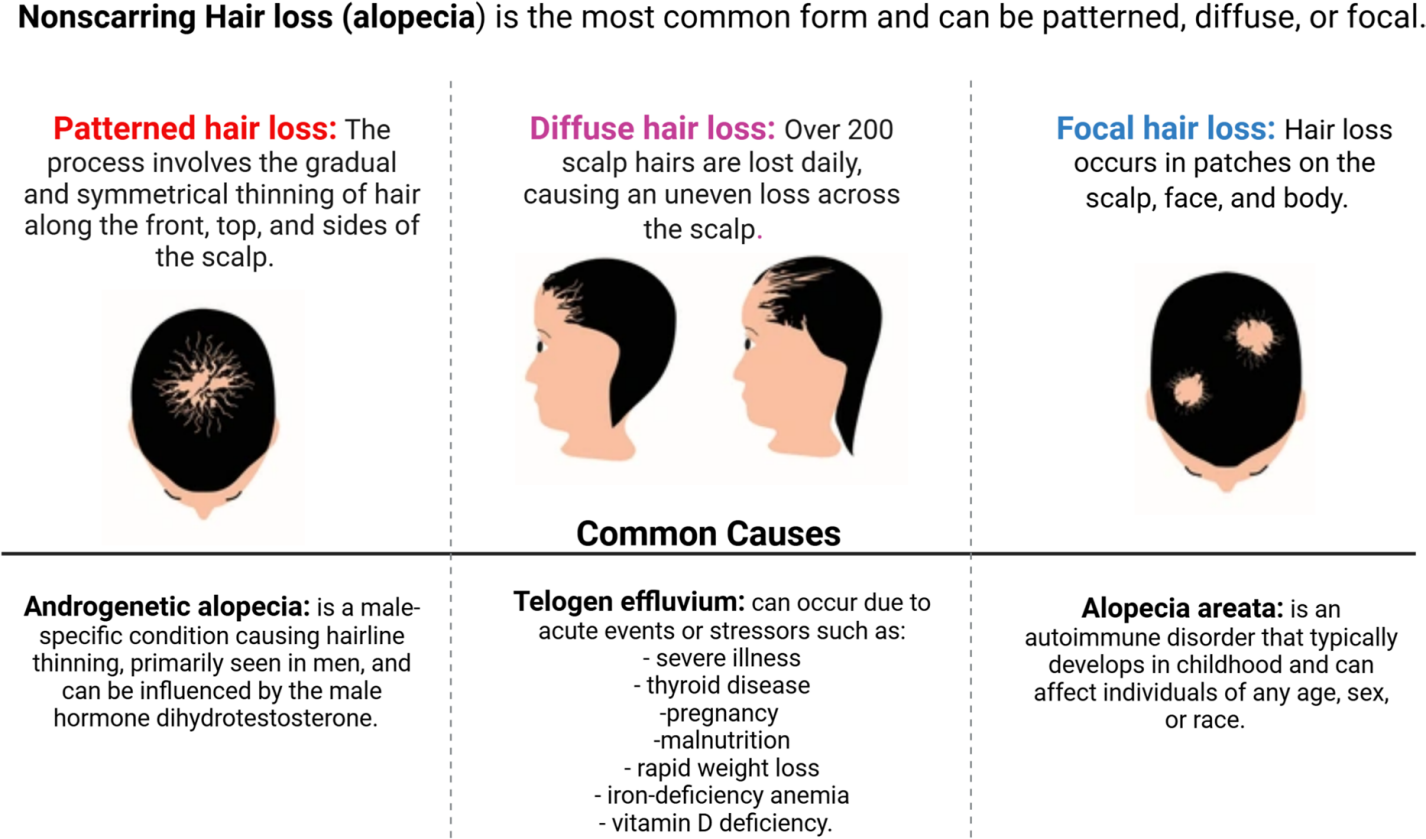
Common causes of hair loss

The pathophysiology of hair loss is a complex process influenced by multiple factors, including genetic predisposition, hormonal changes, immune responses, and environmental influences. Hair follicles undergo a cyclical process comprising three main phases: anagen (growth), catagen (regression), and telogen (resting). Disruption of this cycle can lead to hair loss. For example, in AGA, there is a shortening of the anagen phase and an increase in the proportion of hair follicles in the telogen phase, resulting in thinning and eventual hair loss (Qi and Garza 2014). Numerous factors that impact the hair growth cycle and the condition of hair follicles can cause hair loss. Determining possible treatments and preventative actions requires an understanding of these elements. Genetic predisposition, hormonal fluctuations, illnesses, dietary deficiencies, stress, environmental exposure, hair care routines, and medications are important contributing variables. Different forms of hair loss can result from any of these factors interfering with the hair follicles’ natural activity. It is imperative to address these underlying reasons for hair loss issues to be effectively managed and treated (Fig. 1). Some factors can increase the risk of hair loss, including (Al-Sowayan et al. 2024):

- A family history of balding on the mother’s or father’s side
- Age
- Significant weight loss
- Certain medical conditions, such as diabetes and lupus
- Stress
- Poor nutrition.

#### Genetic factors

The most prevalent cause of hair loss, AGA, is determined by genetic predisposition. It causes baldness patterns and progressive hair thinning in both men and women (Phillips et al. 2017; Gokce et al. 2022; Lin et al. 2016). AGA is a complex genetic condition with a polygenic feature and is commonly known as male-pattern baldness and female-pattern baldness. Changes in the androgen receptor (AR) gene and 5-alpha reductase gene are clinically significant, whereas triple repeat polymorphisms and single nucleotide polymorphisms are associated with androgenic alopecia. Genetic alterations in the WNT signaling pathway regulating dermal papilla cells and androgen metabolism may also precipitate AGA. However, expression and epigenetic studies are limited (Martinez-Jacobo et al. 2018).

This genetic predisposition, a significant factor in hair loss, is influenced by various genetic factors, which can significantly affect hair growth cycles and follicle health. Research indicates that a considerable risk for hair loss might be attributed to genetic factors. According to twin studies, almost 80% of male pattern baldness is heritable (Hagenaars et al. 2017). Given that many people acquire illness from their parents, this suggests a significant genetic component.

Numerous genetic loci have been linked to hair loss by research. More than 250 distinct genetic loci were connected to significant hair loss in a large genome-wide association study (GWAS) that included more than 52,000 men (Hagenaars et al. 2017). The AR gene is critical because its mutations are significantly linked to AGA (Otberg et al. 2007). The AR gene provides instructions for making androgen receptors, which are crucial for the body’s response to androgens like DHT. Hair thinning and loss can result from elevated DHT levels, which can also decrease the anagen period of hair development (Lolli et al. 2017). Hair loss may be exacerbated by genetic differences, leading to more responsive receptors to androgens.

Based on an individual’s genetic makeup, recent research has created polygenic risk scores that can forecast the probability of hair loss. For example, research found that moderate to severe hair loss was substantially more common among those in the top 10% of polygenic scores than in people in the lower percentiles (Hagenaars et al. 2017).

While genetics play a crucial role, environmental factors such as diet, stress, and overall health can also influence hair loss. Hormonal imbalances and nutritional deficits, for instance, can make genetic predispositions worse (Gokce et al. 2022).

#### Hormonal changes

Androgens: Hair follicle shrinkage and hair loss can result from hormonal changes, especially those involving androgens like testosterone and its derivative dihydrotestosterone (DHT). Hormonal imbalances brought on by disorders like women's PCOS can also result in hair thinning (Phillips et al. 2017; Gokce et al. 2022; Lin et al. 2016) (Mounsey and Reed 2009). In addition, thyroid disorders, both hyperthyroidism and hypothyroidism, can lead to hair loss due to hormonal imbalances affecting the hair growth cycle (Phillips et al. 2017; Gokce et al. 2022; Lin et al. 2016).

#### Medical conditions

Autoimmune disorders: Conditions like AA involve the immune system attacking hair follicles, resulting in patchy hair loss (Phillips et al. 2017; Gokce et al. 2022; Lin et al. 2016).

#### Nutritional deficiencies

Dietary influences play a crucial role in the health of hair follicles and the maintenance of hair growth. A balanced diet rich in essential nutrients, including protein, minerals (iron and zinc), and vitamins (especially biotin and vitamin D), is vital for preventing hair loss and promoting hair health. So, their deficiencies can impair hair growth and lead to hair loss, including TE and AGA (Guo and Katta 2017; Gowda et al. 2017). Iron deficiency, for example, can cause hair follicles to receive less oxygen, hindering their activity and increasing shedding. Similarly, zinc deficiency can interfere with the hair development cycle because it is necessary for keratin synthesis and cellular proliferation. Biotin, a B vitamin, is also critical for keratin production, the primary structural protein in hair, whose insufficient levels can result in brittle hair and hair loss. Moreover, omega-3 fatty acids in flaxseeds and fish have anti-inflammatory qualities that may promote hair development and scalp health. Conversely, overconsumption of other nutrients, like vitamin A, can generate hair loss, underscoring the significance of balance and moderation. Furthermore, new evidence indicates that dietary patterns like high-fat and high-sugar diets may render hair loss worse by encouraging hormonal imbalances and inflammation. Therefore, lowering the risk of hair loss and promoting ideal hair development require a well-rounded diet that contains a range of nutrients, especially those proven to enhance hair health (Guo and Katta 2017).

#### Stress

Stress is one of the most common triggers of hair loss issues because it raises the amount of cortisol produced in the body. By degrading hyaluronan and proteoglycans, which integrate components in the extracellular matrix and skin, cortisol has been shown to have a detrimental influence on the hair follicle development mechanism. The primary origin of TE is thought to be both acute and chronic stress. Additionally, stress can exacerbate the forms of alopecia that are mostly brought on by immunological reactions, endocrine imbalances, and toxic causes. Furthermore, hair loss persists due to the stress that follows hair loss; thus, it is a vicious cycle. In animal studies, chronic stress has been linked to perifollicular inflammation, increased mast cell granulation, and hair growth halt. Additional research has also demonstrated that some stress mediators, including cortisol, prolactin, substance P, and adrenocorticotropic hormone, constrain hair growth (Thom 2016).

#### Environmental factors

Toxins and pollution: Exposure to environmental toxins and pollutants can damage hair follicles and contribute to hair loss (Phillips et al. 2017; Gokce et al. 2022; Lin et al. 2016).

#### Hair care practices

Certain hairstyles that pull on the hair (like tight ponytails or braids) can cause traction alopecia. Additionally, harsh hair treatments (such as chemical relaxers or excessive heat styling) can damage hair and lead to its breakage and loss (Phillips et al. 2017; Gokce et al. 2022; Lin et al. 2016).

#### Medications

Some medications, including anti-hypertensives, anti-arrhythmics, statins, anti-metabolites, psychotropic agents, anti-convulsants, anti-coagulants, antiretrovirals, and H_2_ blockers, are among the drugs that cause hair loss as a side effect (Lin et al. 2016). These numerous categories of prescription drugs have been associated with hair loss, presenting it as a potential temporary side effect. It is important to recognize that hair loss is infrequent with these medications, and when it does occur, it may manifest after a few weeks or even years of use. Factors such as dosage, treatment duration, and individual responses play significant roles in the extent of hair loss, if any. Typically, hair growth resumes about 3 to 4 months after discontinuing the medication. Certain cholesterol-lowering drugs, including clofibrate and gemfibrozil (Lopid), may lead to hair loss. Also, some Parkinson’s medications like levodopa (Dopar, Larodopa) and common ulcer medications such as cimetidine (Tagamet), ranitidine (Zantac), and famotidine (Pepcid) have been noted for this side effect. Besides, high blood pressure medications, particularly beta-blockers like atenolol (Tenormin) and metoprolol (Lopressor); common anticoagulants, including warfarin and heparin; and gout medication allopurinol (Zyloprim) occasionally lead to hair loss. Arthritis treatments, such as penicillamine, indomethacin (Indocin), naproxen (Naprosyn), sulindac (Clinoril), and methotrexate, can also contribute to hair thinning. Similarly, excessive doses of vitamin A and its derivatives, including isotretinoin (Accutane), result in hair loss. Nonsteroidal anti-inflammatory drugs (NSAIDs), both over-the-counter options like ibuprofen and prescription variants such as celecoxib (Celebrex), have also been linked to hair loss. Hormone replacement therapy and many oral contraceptives can induce hair thinning in some women, although they are frequently prescribed to combat hair loss in others. Anabolic steroids, often used to enhance muscle mass, can lead to premature baldness in genetically predisposed men, with testosterone and other anabolic hormones being notable examples. Furthermore, while thyroid disorders can cause hair loss, some thyroid medications, such as thiouracil, may also contribute to hair thinning (Patel et al. 2013; Tosti et al. 1994).

**Fig. 2:**
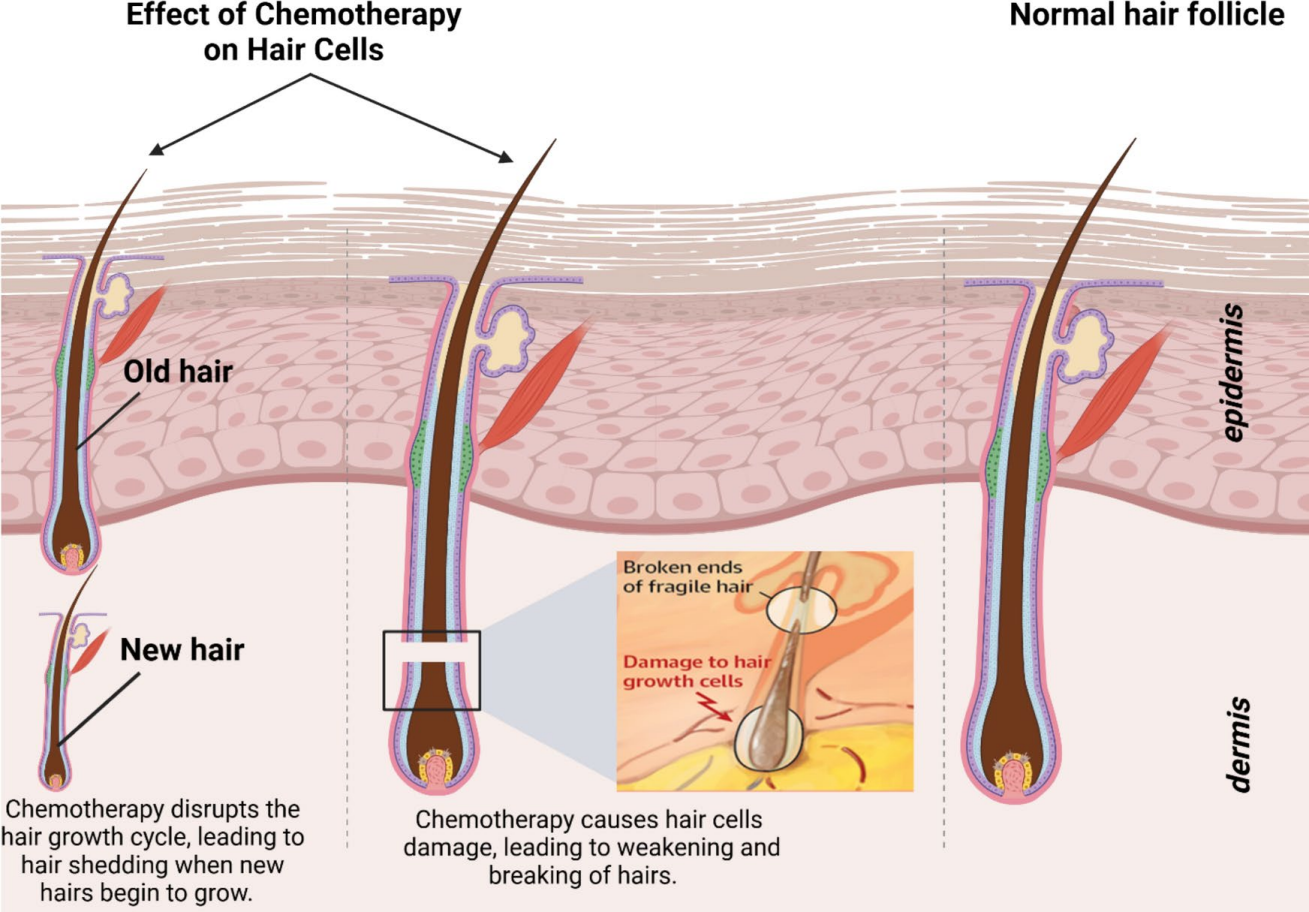
Chemotherapy drugs are used to treat various types of cancer, and hair loss is a common and usually temporary side effect of many of these drugs

**Fig. 3:**
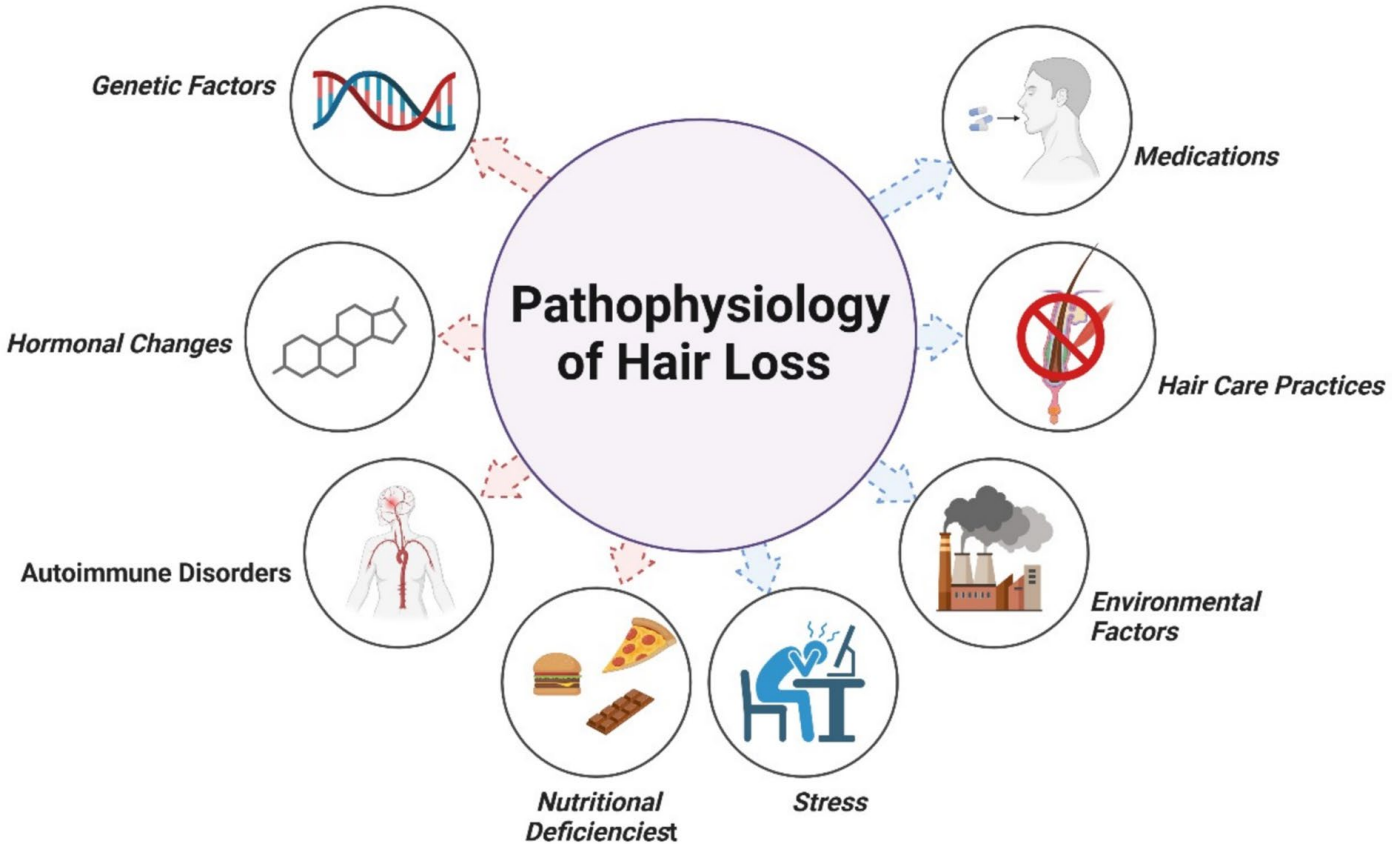
Pathophysiology of hair loss

The incidence of chemotherapy-induced hair loss is estimated to be approximately 65%. However, the prevalence and severity of this condition can vary significantly depending on the specific chemotherapeutic agents and treatment protocols employed. Various classes of anticancer drugs are known to induce alopecia, with the frequency of hair loss differing across the four primary drug classifications (Fig. 2). Antimicrotubule agents, such as paclitaxel, are associated with hair loss rates exceeding 80%. Topoisomerase inhibitors, including doxorubicin, exhibit a 60 to 100% prevalence. Alkylating agents, such as cyclophosphamide, have been linked to hair loss rates greater than 60%. In contrast, antimetabolites, like 5-fluorouracil in combination with leucovorin, present a lower incidence, ranging from 10 to 50%. Furthermore, combination chemotherapy utilizing two or more agents generally results in higher incidences and greater severity of hair loss compared to monotherapy (Coleman 2020). Pathophysiology factors of hair loss are displayed in Fig. 3.

Chemotherapy induces hair loss primarily due to its cytotoxic effects on rapidly dividing cells, including those in the hair follicles. This treatment targets and damages these cells, disrupting the normal hair growth cycle. Specifically, chemotherapy can cause an anagen effluvium, where hair follicles in the anagen (growth) phase abruptly cease mitotic activity. This cessation leads to structural weakness in the hair shaft, resulting in hair shedding that typically begins within 1 to 3 weeks after the initiation of treatment. Various chemotherapeutic agents have different potencies and mechanisms, leading to variability in the severity and pattern of hair loss among patients. Although hair loss is often reversible, patients may experience changes in hair texture or color during regrowth, highlighting the profound impact of chemotherapy on both physical appearance and psychological well-being (Paus et al. 2013).

### Modifiable hair loss risk factors

Hair loss is a regular issue affected by genetics, nutrition, stress, and lifestyle factors (Macklis et al. 2022). While some causes are unavoidable, comprehensive lifestyle modifications can mitigate hair loss, improve hair health, and stimulate regrowth (Null and Feldman 2008).

#### Diet and nutrition

A healthy diet, sufficient to provide the necessary nutrients for optimal healthy hair, is important (Rajendrasingh 2018). Protein is the major constituent of hair keratin, the crucial component for hair strength and integrity via protein intake (Almohanna et al. 2019). Nutrition is a varied account of nutrients and compounds that are stable or not stable (Kinoshita-Ise et al. 2023). Food, such as lean meat, fish, eggs, nuts, and beans, has the high quality of supplying protein (Khaitan et al. 2024). Nutritional deficiencies, particularly ones due to a lack of key vitamins and minerals, can worsen hair loss (Agrawal et al. 2020). The following table (Table 1) elucidates the major functions of each key nutrient contributing to hair growth.

**Table 1:** Vitamins and minerals with their roles in hair health

Vitamin/mineral	Functions and effects on hair	Sources	Ref
Biotin (B7)	Strengthens hair structure, prevents dryness, and promotes growth	Eggs, nuts, seeds, sweet potatoes	Almohanna et al. 2019; Rajput 2018
Vitamin B3 (Niacin)	Reduces inflammation, improves scalp health	Fish, chicken, peanuts, avocados	Iqbal et al. 2019; O'Connor and Goldberg 2021
Vitamin C	Enhances collagen synthesis, antioxidant properties, aids iron absorption	Citrus fruits, bell peppers, broccoli	Gokce et al. 2022; Trüeb and Trüeb 2020a
Vitamin A	Supports cellular differentiation, protects follicles	Carrots, spinach, sweet potatoes	Rushton 2002
Vitamin D	Regulates follicle cycling, forms new follicles	Fatty fish, egg yolks, fortified foods	Ruiz-Tagle, et al. 2018
Vitamin E	Protects follicles from oxidative stress	Almonds, sunflower seeds, spinach	Rajendrasingh 2017
Iron	Prevents anemia, delivers oxygen to follicles	Red meat, spinach, lentils	Park 2015
Zinc	Aids protein synthesis, prevents miniaturization, accelerates hair growth	Oysters, beef, pumpkin seeds	Godswill et al. 2020
Copper	Enhances hair elasticity and pigmentation	Shellfish, nuts, seeds	Haneke and Baran 2010
Selenium	Antioxidant properties, protects against follicular aging	Brazil nuts, seafood	Thompson et al. 2017
Omega-3 fatty acids	Improves elasticity, reduces inflammation, supports scalp health	Fatty fish, chia seeds, walnuts	Muraleedharan et al. 2023
Folic acid (B9)	Aids DNA synthesis, prevents anemia	Leafy greens, beans, fortified cereals	Dattola et al. 2020
Lysine	Promotes hair growth in telogen effluvium	Red meat, eggs, cheese	Singh et al. 2014
Sulfur	Prevents brittle and dry hair	Garlic, onions, broccoli	Singh et al. 2014

#### Stress management

Chronic stress contributes to hair loss, as discussed before, especially telogen effluvium, in which stress changes the hair from the body into the shedding phase (Iglesias et al. 2015). So, stress management techniques are crucial to implement, like meditation and mindfulness, which are practices such as deep breathing exercises and performing mindfulness meditation, reducing cortisol levels and thus affecting hair growth (Trüeb 2015). Yoga and exercise also influence blood circulation positively, bringing nutrients to the hair follicles and reducing stress (Peters et al. 2017). Furthermore, adequate quality sleep modulates hormones of cortisone and melatonin, which, in turn, control hair biology. Targeting 7–9 h of sleep per night promotes general good health (Staufenbiel et al. 2013).

#### Scalp care

Healthy hair begins with a healthy scalp. Proper scalp hygiene in preventive procedures avoids the formation of sebum, dead deskins cells, and residues of products that sebum blocking of hair follicle openings can cause (Fung et al. 2023). Gentle pH-balanced shampoos should be used to clean the scalp without excessive drying (Beauquey 2005). People should also include scalp massage, which increases blood flow and activates the hair follicles (Yu et al. 2006). Hydration is also critical to moisturize the scalp, as dryness can provoke irritation and compromise follicles (Ha et al. 2024).

#### Lifestyle choices

The following are some lifestyle practices that can be beneficial or detrimental to hair health, like avoiding smoking because smoking obstructs blood circulation to the scalp, starving the follicles of critical nutrients, and subjecting them to oxidative stress (Babadjouni et al. 2021). In addition, limiting alcohol consumption as excessive alcohol intake depletes zinc and other vital nutrients (Salomone et al. 2018). Establishing a routine by adhering to regular meal hours, physical activity, and sleep schedule, known as the human bioelectromagnetic system, also maintains the hormonal balance, leading to hair improvement (Afridi et al. 2011).

#### Addressing underlying health issues

Hair loss is frequently a sign of underlying systemic conditions, including hormonal disorders, thyroid dysfunction, or nutrient depletion (Meher et al. 2023). Preventive health examinations and visits with health care practitioners can identify and manage these occult conditions. For instance, they include hypothyroidism, PCOS, and anemia, which commonly trigger hair loss (Khath and San 2024).

### Synthetic drugs for hair loss overview

Hair loss, or alopecia, the common condition affecting millions globally, is caused by various factors such as genetic predisposition, hormonal imbalances, environmental influences, and underlying medical conditions, as discussed before. While herbal treatments have gained attention, synthetic drugs remain the primary approach for hair loss treatment (Youssef et al. 2022; Gasmi et al. 2023; Samra et al. 2024; Cuevas-Diaz Duran et al. 2024). This section critically assesses the major synthetic pharmacological agents used in hair loss management, their mechanisms of action, evidence-based efficacy, side effects, and limitations.

Several synthetic drugs are approved for treating different types of hair loss, particularly AGA and alopecia areata AA. These drugs operate through mechanisms including hormonal modulation, vasodilation, and immunosuppression. Research into novel synthetic treatments continues to evolve, with a growing emphasis on targeted therapies and combination approaches (Vañó-Galván and Park 2024; Pawlik et al. 2024; Sterkens et al. 2021).

#### Finasteride (5α-reductase inhibitor)

Finasteride is an FDA-approved oral medication for male pattern baldness, acting as a type II 5α-reductase inhibitor. It prevents the conversion of testosterone to DHT, thereby reducing follicular miniaturization and extending the hair follicles’ anagen (growth) phase (Bhattacharya et al. 2023; Escamilla-Cruz et al. 2023).

For its efficacy, clinical trials confirm that finasteride increases hair count and density, particularly in the vertex and mid-scalp regions. Long-term use (≥ 2 years) shows sustained hair regrowth in a significant number of users. Some studies suggest that combining finasteride with other topical treatments may enhance efficacy (Shin et al. 2019; Piraccini et al. 2022).

Regarding its side effects, potential adverse effects include sexual dysfunction (e.g., erectile dysfunction, reduced libido, and ejaculation disorders), gynecomastia, and depression. Some patients report post-finasteride syndrome (PFS), characterized by persistent sexual, neurological, and psychological symptoms even after discontinuation (Traish 2020; Fierro et al. 2022).

In terms of its limitations, finasteride is ineffective in women due to its teratogenic potential. Hair regrowth effects are reversed within 6–12 months after stopping treatment. The need for lifelong administration presents a challenge for some users (Mysore and Shashikumar 2016; Iamsumang et al. 2020; Gupta et al. 2022a).

#### Dutasteride (dual 5α-reductase inhibitor)

Dutasteride inhibits type I and type II 5α-reductase isoenzymes, making it more potent than finasteride in reducing DHT levels (Chislett et al. 2023; Gupta et al. 2022b).

Concerning efficacy, studies indicate superior hair regrowth and maintenance compared to finasteride, with notable improvement in AGA patients. Recent research explores its effectiveness in female pattern hair loss, combined with other treatments (Shanshanwal and Dhurat 2017; Almudimeegh et al. 2024).

Concerning the side effects, it is similar to finasteride but with a higher incidence of sexual dysfunction and a longer half-life, leading to prolonged systemic effects. Some studies indicate lower doses may help reduce side effects while maintaining efficacy (Jeh et al. 2019; Estill et al. 2023).

As for the limitations, despite its higher efficacy, dutasteride is not FDA-approved for AGA and is used off-label. The extended half-life raises concerns about prolonged adverse effects, making it less desirable for individuals wary of systemic impact.

#### Minoxidil (potassium channel opener and vasodilator)

Minoxidil is an over-the-counter topical solution or foam used to treat both male and female pattern hair loss. Originally developed as an antihypertensive agent, it was repurposed later for hair regrowth (Randolph and Tosti 2021).

With respect to its efficacy, minoxidil extends the anagen phase and increases hair diameter. Moderate effectiveness is observed, with noticeable improvements in hair density occurring after 4–6 months of consistent use. Studies indicate that combination therapy with microneedling or low-level laser therapy (LLLT) may enhance absorption and effectiveness (Suchonwanit et al. 2019; Zhang et al. 2022; Kaiser et al. 2023).

Regarding its side effects, common adverse events include scalp irritation, dermatitis, unwanted facial hair growth (hypertrichosis), and an initial shedding phase (“minoxidil shedding”). Rare cases of systemic absorption can cause hypotension and tachycardia (Anastassakis 2022; Bhuiyan 2023).

For its limitations, continuous use is required for sustained results; discontinuation results in hair loss within months. It is less effective in advanced hair loss cases. Formulation improvements, such as nanocarrier-based delivery systems, are being investigated to enhance its bioavailability (Stoehr et al. 2019; Nagai et al. 2019).

#### Spironolactone (aldosterone antagonist and anti-androgen)

Spironolactone, a potassium-sparing diuretic with anti-androgenic properties, is prescribed off-label for female pattern hair loss (Wang et al. 2023).

Concerning its efficacy, it is considered effective in reducing hair shedding and improving density in androgen-sensitive alopecia, particularly in women with polycystic ovary syndrome (PCOS). Some studies suggest enhanced efficacy when combined with oral contraceptives (Rajashekar et al. 2022; Burns et al. 2020).

Its side effects and potential adverse effects include hyperkalemia, menstrual irregularities, breast tenderness, fatigue, and dizziness. Newer formulations may help mitigate these issues (deOliveira et al. 2024; Searle et al. 2020).

For its limitations, it is unsuitable for men due to its feminizing effects. Long-term use is necessary for sustained benefits. Research is ongoing into alternative anti-androgen therapies with fewer hormonal side effects (Iszczuk et al. 2024).

#### Corticosteroids (immunosuppressants for AA)

Corticosteroids, available in topical, intralesional, and systemic formulations, are commonly used to suppress autoimmune-mediated hair loss (Parikh et al. 2024; Al Refaei et al. 2023).

Regarding efficacy, it is effective in localized alopecia areata, promoting hair regrowth within weeks. Combination therapy with other immunomodulators may improve outcomes (Evyana et al. 2023).

The side effects include skin atrophy and systemic effects such as weight gain, hypertension, and osteoporosis with prolonged use. Newer delivery methods aim to reduce these risks (Lim and Bolster 2019).

For the limitations, high relapse rates are common, particularly after stopping treatment. Long-term systemic use is discouraged due to the risk of hormonal imbalances, other complications, and severe adverse effects (Suchonwanit et al. 2022; Chanprapaph et al. 2022).

### Herbal medicine for hair loss history

Managing alopecia without adverse effects remains a problem for medical professionals. Herbal medicines are still widely used in many countries for historical and cultural reasons despite the availability of modern medicine. Men and women have been looking for solutions to hair loss since the dawn of recorded history. The oldest known prescription dates back 5000 years and was for hair loss (Panagotacos 2024). This section documented the most common historically used herbal remedies for hair loss, as illustrated in this table (Table 2).

**Table 2:** Historical use of herbal medicine for hair loss

Region/people	Herbal method	Comment	Ref
Ayurveda (India)	**Bhringraj (*Eclipta prostrata*)**: Applied as oil or paste to scalp **Amla**: Consumed or used in hair oils **Shikakai**: Used as a natural shampoo	Bhringraj is called the “King of Hair” for its rejuvenating effects. Amla strengthens hair and combats dandruff. Shikakai cleanses without stripping natural oils	Pallavi et al. 2023; Bansode et al. n.d.; More and Somani n.d.
Traditional Chinese Medicine (China)	**He Shou Wu (Fo-ti)**: Consumed as a tonic **Ginseng**: Applied topically or taken orally **Angelica Root**: Included in hair tonics	He Shou Wu is believed to restore natural hair color and balance “yin” energy. Ginseng stimulates circulation, strengthening hair roots	Trüeb and Trüeb 2020b; Waltz 2004
Middle Eastern Practices (Arabs)	**Black Seed Oil (*Nigella sativa*)**: Applied directly or mixed with honey **Fenugreek Seeds**: Soaked and made into a paste for the scalp **Henna**: Used as a natural dye and scalp conditioner	Black seed oil is known for its antimicrobial and anti-inflammatory properties. Fenugreek is believed to strengthen roots and reduce hair fall. Henna conditions hair while coloring it naturally	Bakhru 1992; Mariod et al. 2017
Ancient Egyptians	**Castor Oil**: Applied to hair and scalp **Aloe Vera**: Used as a scalp soother **Fenugreek**: Used to strengthen hair	Castor oil was considered essential for thickening hair. Aloe vera was prized for its hydrating and regenerative properties. Fenugreek was valued for its ability to reduce hair thinning	Dweck n.d.; Aburjai and Natsheh 2003
European Traditions	**Rosemary Oil**: Massaged into the scalp **Nettle Leaf**: Used in rinses or consumed as tea **Horsetail Extract**: Used in hair tonics	Rosemary oil improves scalp circulation. Nettle leaf, rich in silica, strengthens hair. Horsetail extract fortifies hair strands	Franklin 2024; Ellis 2015
African Practices	**Shea Butter**: Applied to hair as a moisturizer **Aloe Vera**: Used as a gel or juice for scalp hydration **Clays and Natural Oils**: Used for detoxifying and nourishing the scalp	Shea butter protects against environmental damage, and aloe vera soothes and promotes growth by regenerating cells	Keller 1999; Griffin 2012
Native Americans	**Yucca Root**: Used as a natural cleanser **Aloe Vera**: Applied for scalp hydration and healing	Yucca root cleanses while protecting scalp health. Aloe vera encourages hair growth through its hydrating and regenerative properties	Simons 2013; Gage 1996

As elaborated, there are drug-based therapies for alopecia that include minoxidil and finasteride, but these have been linked to a variety of negative effects in patients. As a result, novel therapeutic approaches based on bioactive compounds have been promoted in order to lessen the risk of anti-hair-loss drugs (Park and Lee 2021).

#### Herbal medicine preclinical data

Natural products containing plant remedies have been used for thousands of years and shown to be safe with little adverse effects. Several studies have demonstrated that plants and their extracts can boost hair growth. Furthermore, commercial medications containing these natural substances have been created to treat alopecia. Several clinical, animal, and cell research have been conducted to investigate the anti-alopecia properties of plant-derived biochemicals. These preclinical studies of phytochemicals with anti-alopecia effects are summarized in Table 3.

**Table 3:** Preclinical studies of key herbal remedies for hair loss

Name of plant	Family	Type of extract	Part used	Methods	Results	Ref
*Ginkgo biloba*	(Ginkgoaceae)	Essential oil	Leaves	In vivo	Hair growth activation	Kolekar et al. 2021; Zgonc Škulj et al. 2020
*Camellia sinensis* (green tea)	Theacea	Ethanolic extract	leaves	In vivo	It inhibits the activity of 5α-reductase, which results in a decreased DHT formation	Zgonc Škulj et al. 2020
*Polygonum multiflorum*	(Polygonaceae)	Water extract	Roots, leaves	In vivo	Promote hair growth	Shen et al. 2018b; Park et al. 2011a; Li et al. 2015
*Chamaecyparis obtusa*	Cupressaceae	Essential oil	Whole plant	In vivo	Promote hair growth	Eun et al. 2010
*Cuscuta reflexa*	Convolvulaceae	Petroleum ether or ethanol extract	Stem	In vivo In vitro	Promote hair growth Upregulate testosterone level	Patel et al. 2014
*Eclipta alba*	Compositae	Methanol extract	Whole plant	In vivo	Promote hair growth	Datta et al. 2009
*Fructus panax ginseng*	Araliaceae	Ethanol extract	Root	In vivo In vitro	Promote hair growth Promote proliferation and inhibit apoptosis of human DPCs	Park et al. 2011b
*Eclipta alba*	Compositae	Petroleum ether extract	Aerial part	In vivo	Promote the proliferation of HaCaT	Begum et al. 2015
*Zizyphus jujuba*	Rhamnaceae	Essential oi	Seed	In vivo	Promote hair growth	Yoon et al. 2010
*Aconite Ciliare*	Ranunculaceae	Water extract	Root tuber	In vivo In vitro	Promote hair growth. Promote the proliferation of human DPCs	Park et al. 2012
*Asiasari radix*	Aristolochiaceae	Ethanol extract	Root	In vivo In vitro	Promote hair growth. Promote the proliferation of HaCaT and human DPCs	Roh et al. 2002
*Panax ginseng*	Araliaceae	70% ethanol extract	Root	In vivo In vitro	Promote human follicles growth Promote proliferation and inhibit apoptosis of ORS	Lee et al. 2017
*Polygonum multiflorum* (Preparata)	Polygonaceae	Water extract	Root	In vivo	Promote hair growth	Shen et al. 2018b
*Carthamus tinctorius*	Asteraceae	Ethanol extract	Floret	In vivo In vitro	Promote hair growth Promote the proliferation of both DPCs and HaCaT	Junlatat and Sripanidkulchai 2014
*Phyllanthus emblica*	Phyllanthus emblica	Ethanol extract	Fruit	In vivo	Promote hair growth	Kumar et al. 2012
*Rosmarinus officinalis*	Lamiaceae	50% ethanol extract	Leaf	In vivo In vitro	Promote hair growth Promote the proliferation of DPCs	Murata et al. 2013
*Pueraria thomsonii*	Leguminosae	50% ethanol extract	Flower	In vivo	Promote hair growth	Murata et al. 2012
*Pueraria lobata*	Leguminosae	Ethanol extract	Root	In vivo	Promote hair growth	Murata et al. 2012
*Erica multiflora*	Ericaceae	Ethanol extract	Whole plant	In vivo In vitro	Promote hair growth Promote the proliferation of DPCs	Kawano et al. 2009
*Thuja orientalis*	Cupressaceae	Hot water extract	Leaf	In vivo	Promote hair growth	Zhang et al. 2013
*Ecklonia cava*	Lessoniaceae	Methanol extract	Whole plant	In vivo In vitro	Promote hair growth Promote the proliferation of ORS and DPCs and elongation of human hair shaft	Bak et al. 2013
*Ecklonia cava*	Lessoniaceae	Enzymatic extract	Whole plant	In vivo In vitro	Promote hair growth Promote the proliferation of human DPCs	Kang et al. 2012
*Chrysanthemum zawadskii*	Asteraceae	Methanol extract	Whole plant	In vivo	Promote hair growth	Begum et al. 2015
*Chrysanthemum zawadskii*	Asteraceae	70% ethanol extract	Whole plant	In vivo	Promote hair growth	Li et al. 2014
*Platycarya strobilacea*	Juglandaceae	Methanol extract	Whole plant	In vivo	Promote hair growth	Kim et al. 2014
*Sophora flavescens*	Leguminosae	Water extract	Root	In vitro	Promote the proliferation of HaCaT and elongation of human hair follicles	Takahashi et al. 2016
*Schisandra nigra*	Schisandraceae	85% ethanol extract	Fruit	In vivo In vitro	Promote hair growth Promote the proliferation of DPCs	Kang et al. 2009
*Alpinia zerumbet*	Zingiberaceae	Ethyl acetate and butanol extract	Leaf	In vitro	Promote the proliferation of hair cells	Taira et al. 2017
*Geranium sibiricum*	Geraniaceae	Methanol extract	Whole plant	In vivo In vitro	Upregulate IGF-1, VEGF, HGF, SCF, ki-67 expression downregulates TGF-β and the number of mast cells	Boisvert et al. 2017
*Delphinium staphisagria*	Ranunculaceae	Vinegar and water extract	Seed	In vitro	Promote proliferation of HaCaT and human endothelial cells	Koparal and Bostancıoğlu 2016
*Rumex japonicus*	Polygonaceae	95% ethanol extract	Root	In vivo In vitro	Promote hair growth Induced antiapoptsis and proliferation of DPCs and HaCaT	Lee et al. 2016
*Platycladus orientalis*	Cupressaceae	75% ethanol extract	Leaf	In vivo	Promote hair growth	Zhang, et al. 2016
*Acanthopanax koreanum*	Araliaceae	Methanol extract	Leaf	In vitro	Promote the proliferation of DPCs	Kim et al. 2012
*Citrullus colocynthis*	Cucurbitaceae	Petroleum ether extract	Fruit	In vivo	Promote hair growth	Dhanotia et al. 2011
*Crataegus pinnatifida*	Rosaceae	Water extract	Fruit	In vivo In vitro	Promote hair growth Promote the proliferation of DPCs	Shin et al. 2013
*Allium tuberosum Rottler ex Spreng*	Liliaceae	Ethanol extract	Whole plant	In vivo	Promote hair growth	Park et al. 2015
*Sargassum muticum*	Sargassaceae	80% methanol extract	Whole plant	In vivo In vitro	Promote anagen initiation Promote the proliferation of DPCs	Kang et al. 2016
*Malva verticillata*	Malvaceae	Ethanol extract	Seed	In vitro	Promote the proliferation of DPC	Jang et al. 2016
*Lycopersicon esculentum*	Solanaceae	Ethyl acetate extract	Fruit	In vivo	Promote hair growth	Choi et al. 2013
*Glycyrrhiza glabra*	Fabaceae	Petroleum ether extract	Root	In vivo	Promote hair growth	Upadhyay et al. 2012
*Centella asiatica*	Apiaceae	Titrated extract	Whole plant	In vitro	Promote the proliferation of human DPCs	Choi et al. 2017

### Clinical trials and evidence-based research on phytotherapy for hair loss

Hair loss, regardless of its underlying cause, can significantly impact an individual’s self-esteem and quality of life. While conventional treatments, such as minoxidil and finasteride, exist, they can be associated with undesirable side effects and may not be convenient for all individuals. This has driven growing interest in phytotherapy, utilizing plant-based extracts for medicinal purposes as a potential alternative or adjunctive treatment for hair loss. Phytotherapy offers several advantages that make it an attractive option for hair loss management, owing to its natural origin. Many people prefer plant-based remedies due to their recognized safety and alignment with holistic health practices. Additionally, when used appropriately, plant extracts often have a milder side effect profile than synthetic drugs and offer a potentially safer profile than other pharmacological options. These extracts also contain a complex array of bioactive compounds that can target various aspects of hair loss pathogenesis, such as inflammation, hormonal imbalances, and oxidative stress, posing multiple mechanisms of action in the treatment plan.

Clinical trials have begun to shed light on the potential benefits of phytotherapy in managing hair loss caused by various causes. These trials have explored the use of a wide variety of plant extracts, including fenugreek seed extract, to investigate its effects on hair loss in women with PCOS, rice berry by-products, and Thai herbal extracts to evaluate their efficacy as hair tonics in reducing hair loss, grey hair, and dandruff. Furthermore, a blend of botanicals, vitamins, and minerals is formulated as an herbal shampoo or solution to test their efficacy in treating AGA or TE. These formulations were compared to the 5% and 3% minoxidil solution for managing AGA. Rosemary essential oil was also compared to 2% minoxidil for AGA treatment. The following sections will delve deeper into the specific findings of these clinical trials and explore the potential of phytotherapy in managing different types of hair loss, including AGA, TE, AA, and hair loss associated with certain medical conditions or medications.

### Methodology

The primary objective of this systematic review is to evaluate the efficacy and safety of phytotherapy in hair loss management. This review encompasses various etiologies, including AGA, AA, TE, and hair loss associated with hormonal imbalances such as polycystic ovary syndrome (PCOS) and hypothyroidism, as well as chemotherapy-induced alopecia (CIA). It also focuses on identifying effective herbal interventions, assessing their comparative efficacy against standard pharmacological therapies, and exploring their role in improving patient outcomes by synthesizing evidence from randomized clinical trials (RCTs). The review adheres to the Preferred Reporting Items for Systematic Reviews and Meta-Analyses (PRISMA) guidelines, ensuring methodological transparency and rigor. In addition to the clinical studies and evidence-based research on herbal medicine for hair loss that form the core of this systematic review, other complementary sections were included to provide a comprehensive understanding of the topic, though these were not systematically searched. These sections address key contextual elements, including the deep pharmacology of hair loss, its pathophysiology, contributing factors, and associated risk factors such as genetic predisposition and dietary influences. The limitations of synthetic drugs and their side effects, as well as lifestyle modifications that can impact hair loss, were also considered. Moreover, this review discusses the historical use of herbal medicine for hair loss, key herbal remedies (including essential oils, extracts, and pure compounds), and therapeutic applications and formulations. These additional components provide indispensable background knowledge to support the systematic review findings and offer a broader perspective on the role of phytotherapy in treating hair loss. However, a systematic search strategy was employed for the mentioned supplementary sections to ensure an extensive literature review.

#### Search strategy

A comprehensive search strategy was accurately designed to systematically identify studies relevant to the role of phytotherapy in hair loss treatment. The clinical trials’ search was conducted over 2 weeks, from December 15, 2024, to December 28, 2024, and covered multiple electronic databases and clinical trial registries. These resources were chosen to ensure an inclusive coverage of relevant literature and a focused retrieval of high-quality, peer-reviewed studies and ongoing trials with primary published results. The electronic databases included PubMed, Cochrane CENTRAL, Embase, Web of Science (WOS), and Scopus. They were selected for their complementary strengths in delivering comprehensive, peer-reviewed biomedical research, rigorous evidence from RCTs, extensive indexing of pharmacological studies, and profound multidisciplinary coverage, ensuring a thorough compilation of literature on phytotherapy and hair loss. The search was not only confined to these databases but also integrated clinical trial registries, like the National Institutes of Health (NIH) and the World Health Organization (WHO). The ClinicalTrials.gov and WHO International Clinical Trials Registry Platform (ICTRP) were accessed to get significant information on the ongoing and completed clinical trials, guaranteeing the incorporation of emerging, unpublished, and international trial data. The deployed search strategy targeted to capture a robust and complete evidence base for this review by leveraging the strengths of these databases and registries.

The clinical trials’ search strategy used a combination of Keywords and Medical Subject Headings (MeSH) terms to ensure the precise identification of studies relevant to hair loss, phytotherapy, and associated clinical trials. To optimize the sensitivity and specificity across multiple databases, an iterative approach was implemented to refine the search strings, ensuring comprehensive coverage while minimizing irrelevant results. Consequently, the search strategy was systematically developed around three main concepts: Population, Intervention, and Study Design.

For the population, the search included terms encompassing a wide range of hair loss conditions. General terms such as “hair loss,” “alopecia,” and “hair fall” were combined with more specific conditions like “androgenetic alopecia,” “alopecia areata,” “telogen effluvium,” “female pattern hair loss,” and “male pattern hair loss.” Broader descriptors, including “bald,” “baldness,” and “balding,” were also incorporated to ensure that no relevant studies were excluded based on variations in terminology. This comprehensive approach aimed to capture studies addressing the various forms and contexts in which hair loss occurs.

The search focused on phytotherapy and related plant-based treatments, incorporating a wide range of keywords to ensure inclusivity for the intervention. Common terms such as “phytotherapy,” “herbal medicine,” and “natural remedies” were used alongside specific descriptors, including “botanical interventions,” “plant-based medicine,” “medicinal plants,” “botanical extracts,” and “herbal extracts.” Additionally, wide-ranging terms such as “natural treatments,” “natural products,” and “complementary therapies” were included to cover various approaches to plant-based interventions for hair loss. These terms allowed the search to gather diverse formulations and methodologies associated with phytotherapeutic interventions.

Regarding the study design, the search emphasized rigorous methodological approaches by including terms related to clinical trials. These included “randomized controlled trial,” “RCT,” “controlled study,” “interventional study,” “prospective trial,” and “human study.” Including these terms ensured that the search results were focused on high-quality evidence from studies designed to provide reliable insights into the efficacy and safety of phytotherapy for hair loss.

These search strings were primarily utilized for electronic databases such as PubMed, Cochrane CENTRAL, WOS, Scopus, and Embase. While for searches conducted in clinical trial registries, such as ClinicalTrials.gov and WHO ICTRP, a more directed approach was adopted. Instead of full search strings, simple keywords were applied to align with the functionality of these platforms. Terms like “hair loss” were used in the condition/topic field, and “herbal medicine” was entered under the intervention field. The search was also filtered to include studies with reported results in the data provided by these registries to obtain trials with available outcomes. This previously mentioned strategy, with its detailed utilized search strings, is provided in the supplementary materials in the appendix.

Moreover, several filters were applied during the search phase to ensure the relevance and quality of the studies included in this systematic review. First, a language filter was implemented, limiting the selection to studies published in English. This decision was made to ensure consistency in interpretation and analysis, as including non-English studies could introduce variability in understanding and data extraction. Second, a study-type filter was applied to focus exclusively on high-quality evidence. The search was restricted to RCTs, interventional studies, and other human studies, as these designs provide the most reliable data for evaluating the efficacy and safety of phytotherapy in hair loss management, unlike animal studies used to introduce mechanistic investigations. A date range filter was also used to include studies published between January 2014 and December 2024. This 10-year window was chosen to prioritize recent and clinically relevant findings, reflecting advancements in phytotherapy research and confirming that the evidence aligns with current practices. Lastly, the review excluded grey literature and focused solely on peer-reviewed articles and scholarly reviewed publications to build a strong and credible evidence base, minimizing the risk of bias and enhancing the reliability of the findings. These filters collectively certified that the studies specified for inclusion were methodologically consistent, clinically relevant, and of the highest possible quality.

While the clinical trials section adhered strictly to the systematic search strategy, other sections of this review, such as those exploring the pharmacology, pathophysiology, and historical use of herbal medicine, relied on broader, non-systematic methods to gather background information. The search process for these sections utilized several keywords, including “herbal medicine,” “hair loss,” “alopecia,” “natural remedies,” and “hair growth promotion.” Various databases, like PubMed, Scopus, WOS, and Google Scholar, were also utilized. The review systematically captured a robust body of evidence, providing relevance and methodological consistency across the included studies by employing this comprehensive and tailored search. This methodological approach strengthens the reliability of the findings and highlights the review’s commitment to addressing the research question comprehensively.

#### Study selection

The study selection process for this systematic review was conducted as a two-stage approach to ensure that only relevant and high-quality studies were included in the final analysis. The initial stage involved screening the titles and abstracts of all retrieved records against predefined inclusion criteria using Rayyan AI, a web-based automated screening tool. This step intended to identify potentially relevant studies while excluding those clearly outside the scope of the review. Studies deemed unclear or categorized as “maybe” were proceeded to the next stage for further evaluation. In the second screening stage, full texts of potentially eligible studies were thoroughly assessed for inclusion, with decisions guided by the predetermined inclusion and exclusion criteria. Any discrepancies in inclusion decisions were resolved through detailed discussions between the independent reviewers to reach a consensus, ensuring an unbiased selection process.

The criteria for this selection were designed to focus on studies that evaluated the efficacy and safety of phytotherapy interventions for hair loss across the five PICOS elements: Participants/Population (P), Intervention (I), Comparators/Control (C), Outcomes (O), and Study Design (S).

For the population, the review included studies involving adults and children diagnosed with various forms of scalp hair loss. These included AGA (male or female pattern baldness), AA, TE, anagen effluvium (e.g., chemotherapy-induced hair loss), and hair loss associated with hormonal imbalances such as PCOS and hypothyroidism. However, studies were excluded if they focused on hair loss due to scalp infections or infestations, hair loss on areas other than the scalp (e.g., beard), forced hair loss such as traction alopecia, or scarring forms of hair loss, including cicatricial alopecia and frontal fibrosing alopecia. This targeted approach ensured that the population studied was representative of conditions most relevant to the usage of phytotherapy interventions.

For the intervention, eligible studies evaluated phytotherapy, defined as plant-based or botanical treatments. These included topical or oral formulations of medicinal plants, plant-derived compounds such as essential oils, botanical extracts, and decoctions, along with traditional remedies used in Traditional Chinese Medicine. Both single and combination phytotherapeutic agents were also included. Studies were excluded if they involved combined phytotherapy and conventional pharmacological treatments without the capability to isolate phytotherapy’s specific effects. Additionally, studies examining other complementary or non-phytotherapy medicines, such as Ayurveda, marine-based compounds, music therapy, laser red light therapy (phototherapy), or platelet-rich plasma therapy, were excluded as these fell outside the scope of the review.

For the comparators, eligible studies included a control arm (placebo or no treatment) or comparisons with standard pharmacological therapies for hair loss, such as minoxidil or finasteride. Non-phytotherapeutic complementary interventions were also qualified as comparators, provided that the effects of these interventions could be separated from those of phytotherapy. Studies that lacked a comparator group were excluded, as they did not allow for the evaluation of the relative efficacy or safety of phytotherapy interventions.

For the outcomes, the review prioritized studies that reported both primary and secondary outcomes. Primary outcomes included objective measures such as hair density (assessed by trichoscopy or equivalent visualization techniques), total hair count (per cm^2^), hair growth rates (length, thickness, and volumetric changes) supported by scores or global photographs, and reductions in hair shedding. Secondary outcomes encompassed patient-reported measures, including satisfaction questionnaires and quality of life, along with additional objective metrics, such as alopecia patch size, vellus and terminal hair count, and anagen-to-telogen hair ratios. Safety outcomes, which included the occurrence of adverse events and changes in hormonal biomarkers (e.g., androgen levels in PCOS or thyroid function markers), were also deemed essential inclusion.

For the study design, the review exclusively included RCTs to ensure the highest level of evidence for assessing the efficacy and safety of phytotherapy interventions. Observational studies, such as cohort, case–control, and cross-sectional studies, as well as non-randomized interventional trials like quasi-experimental studies, were excluded. In addition, animal studies, narrative reviews, and non-comparative studies were excluded, as these designs do not provide the rigorous evidence necessary for this review’s objectives.

This systematic and rigorous approach to study selection ensured that only the most relevant and methodologically complete studies were included in the review, providing a well-established foundation for evaluating the function of phytotherapy in hair loss management.

#### PRISMA framework

**Fig. 4:**
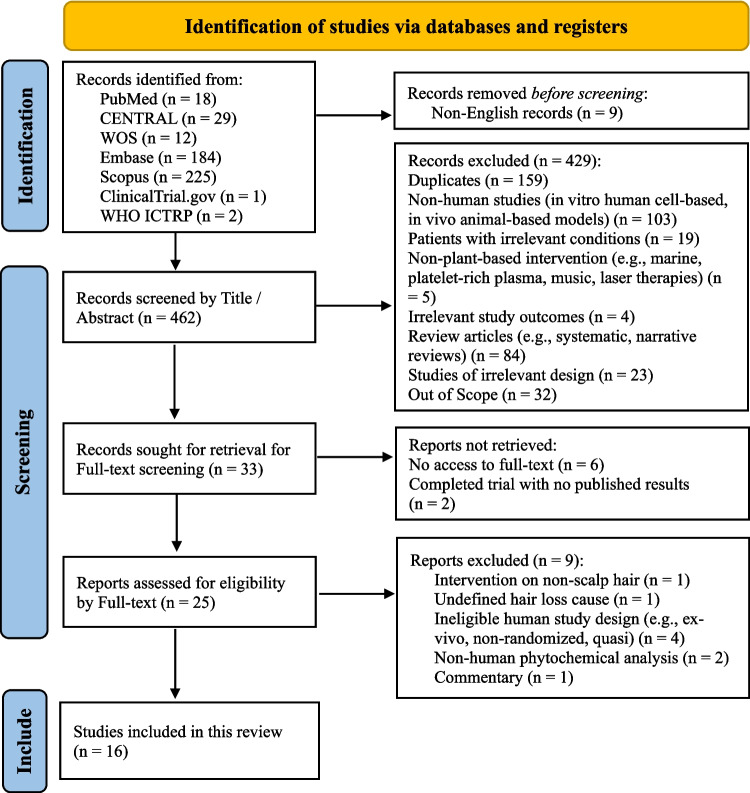
PRISMA flow diagram

The study selection process for this systematic review was thoroughly documented using the PRISMA flow diagram. This approach ensured transparency, methodological precision, and reproducibility, detailing each stage of study identification, screening, eligibility assessment, and final inclusion. Besides, the flow diagram (Fig. 4) provided a clear rationale for study exclusions, particularly during the full-text screening phase.

The identification phase yielded a total of 471 records from multiple databases, including PubMed (*n* = 18), CENTRAL (*n* = 29), Web of Science (*n* = 12), Embase (*n* = 184), Scopus (*n* = 225), ClinicalTrials.gov (*n* = 1), and WHO ICTRP (*n* = 2). To align with the language inclusion criteria of the review, nine non-English records were excluded before screening, whereas CENTRAL (1), Web of Science (2), Scopus (3), and Embase (3). Notably, the initial search in Scopus returned approximately 40,000 records. This excessive and impractical volume of results was tackled by applying additional filters that limited the search to research articles and restricted it to title and abstract fields instead of all paper fields, as done in the other databases. This refinement reduced the Scopus results to 225 relevant records, allowing for feasible screening and consistency with the methodology applied to the other databases.

The screening phase was conducted on 462 records after removing duplicates and non-English studies. These records were assessed based on their titles and abstracts, resulting in the exclusion of 429 records for various reasons. These included 159 duplicate studies, 103 non-human research such as in vitro or animal-based models, 19 irrelevant patient conditions, five non-plant-based interventions (e.g., marine therapies, platelet-rich plasma, music, or laser treatments), four irrelevant study outcomes, 84 review articles (systematic or narrative reviews), 23 irrelevant study designs (e.g., case reports), and 32 studies outside the scope of the review. Following this meticulous screening process, 33 records were deemed eligible for full-text retrieval.

Eight records could not be retrieved from the 33 sought for full-text assessment. This included six studies for which full-text access was unavailable and two completed trials that had not published their results yet. Consequently, 25 studies were fully evaluated, resulting in the exclusion of nine studies for the following reasons: (1) interventions targeting non-scalp hair, (1) undefined causes of hair loss, (4) ineligible human study designs such as ex vivo, non-randomized, or quasi-experimental designs, (2) non-human phytochemical analysis, and (1) commentary papers. Ultimately, 16 studies satisfied the predefined inclusion criteria and were included in the qualitative synthesis. These studies formed the foundation for evaluating the efficacy and safety of phytotherapy interventions for hair loss.

The following PRISMA flow diagram (Fig. 4) documents the thorough previously explained study selection stages. The detailed account of the identification, screening, and eligibility phases illustrates the rigor of the methodology, while the inclusion and exclusion criteria establish a strong framework for selecting relevant studies. This comprehensive approach creates a reliable dataset for subsequent qualitative and quantitative analysis, ensuring the validity of the review’s findings.

#### Data extraction

The data extraction stage was meticulously conducted using a predefined, standardized data extraction form created in Microsoft Excel. This form was developed to ensure consistency, accuracy, and comprehensiveness in obtaining all relevant details from the included studies. The extracted data included key study characteristics, population demographics, intervention specifics, comparator details, outcome measures, and overall conclusions, providing a detailed foundation for analysis and synthesis.

For the study characteristics, information was extracted on each study’s aim and objective, authors, year of publication, country, clinical setting, and funding source to provide contextual and methodological insights. Details about the study duration were also recorded, allowing an evaluation of whether the timeframe was sufficient to assess the efficacy and safety of the phytotherapeutic interventions.

Regarding the population characteristics, comprehensive data about the study population were collected, including the total sample size, the number of participants who dropped out, and the distribution of participants across study arms. Key demographic data, such as age, gender, and the specific cause of hair loss (e.g., androgenetic alopecia, telogen effluvium, or chemotherapy-induced hair loss), were noted. Inclusion and exclusion criteria were also documented to understand the population’s characteristics and guarantee comparability across studies.

Concerning the intervention details, extensive information on the interventions was extracted to capture the diversity of phytotherapy approaches used. This included the plant species employed, preparation methods (e.g., decoctions, extracts, or essential oils), dosage, route of administration (e.g., topical or oral), treatment duration, and frequency of application. These details were critical for identifying potential variations in efficacy and exploring the mechanisms underlying the interventions’ effects.

As for the comparator/control details, the same level of detail as for the intervention arm was extracted for the comparator or control group. This included information on the type of comparator (e.g., placebo or standard pharmacological treatments such as minoxidil) and the methodology used to ensure consistency and fairness in the comparison.

Respecting the outcome domains and measures, data were collected on all outcome domains, including primary and secondary outcomes. These included measures such as hair density, hair count, hair growth rates, patient-reported satisfaction, and quality of life. Time points for outcome assessment and the tools used for measurement (e.g., trichoscopy, photographic evidence) were also recorded to evaluate the robustness and reliability of the reported findings.

About the conclusions, the key findings of each study were extracted to provide a clear understanding of the overall evidence. These data were essential for summarizing the results and drawing meaningful conclusions about the efficacy and safety of phytotherapy in hair loss treatment.

The data extracted using the Excel-based form enabled the creation of an expanded summary of findings (SoF) table, provided in the supplementary material in the appendix for detailed reference. A shortened version of the SoF table, specifically focusing on clinical trials, is presented in Table 6 of the clinical trials section. This structured and standardized data extraction process ensured that all relevant information was acquired systematically, facilitating a rigorous and transparent evidence synthesis.

#### Risk of bias assessment

The risk of bias assessment for the included RCTs was conducted using the Cochrane Risk of Bias 2 (ROB2) tool, specifically the current version of August 22, 2019. This revised tool is widely recognized for its systematic and robust evaluation of potential biases in clinical trials, ensuring the reliability and validity of the synthesized evidence. The assessment process was guided by the ROB2 crib sheet, summarizing the proper use of the tool, and implemented using an Excel workbook containing macros (ROB2_IRPG_beta_v9, 9 th version) designed to streamline the risk-of-bias evaluation.

The ROB2 tool evaluates five key domains of bias: (1) bias arising from the randomization process, (2) bias due to deviations from intended interventions, (3) bias due to missing outcome data, (4) bias in the measurement of the outcome, and (5) bias in the selection of the reported result. The first domain assesses whether the randomization sequence was adequately generated and concealed to prevent selection bias. The second domain analyzes whether participants were exposed to unintended interventions or deviations that might have influenced the study outcomes. The third domain considers the completeness of outcome data and assesses whether missing data might have impacted the reported results. The fourth domain examines the reliability and objectivity of outcome measurements, including whether assessors were blinded to treatment allocation. Finally, the fifth domain estimates the risk of selective outcome reporting, ensuring all pre-specified outcomes were reported transparently. Each domain was rated as “low risk,” “some concerns,” or “high risk” based on the ROB2 criteria, and these judgments were synthesized into an overall risk-of-bias rating for each study.

**Fig. 5:**
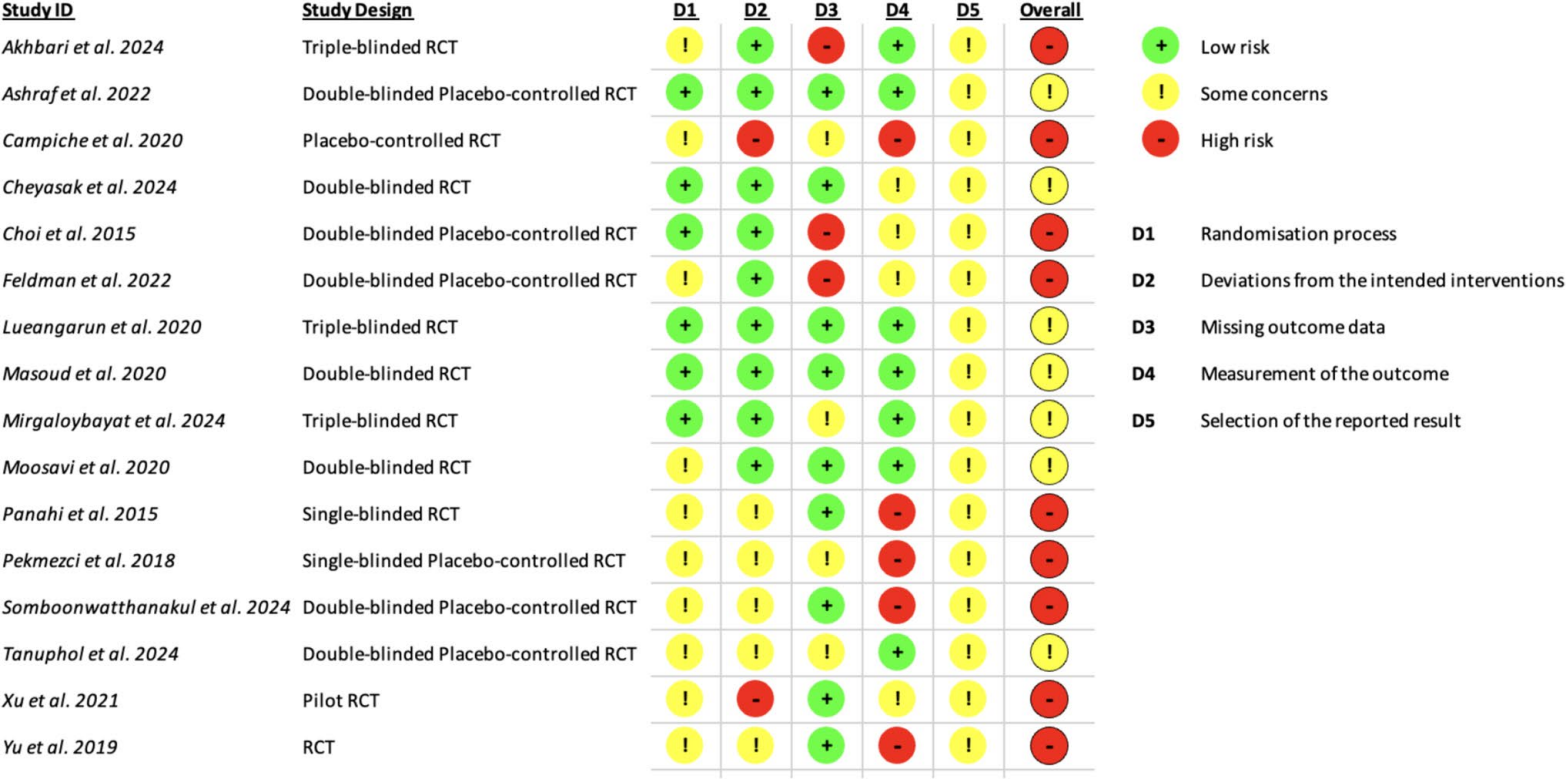
Risk of bias assessment using ROB-2 tool for each clinical trial

**Fig. 6:**
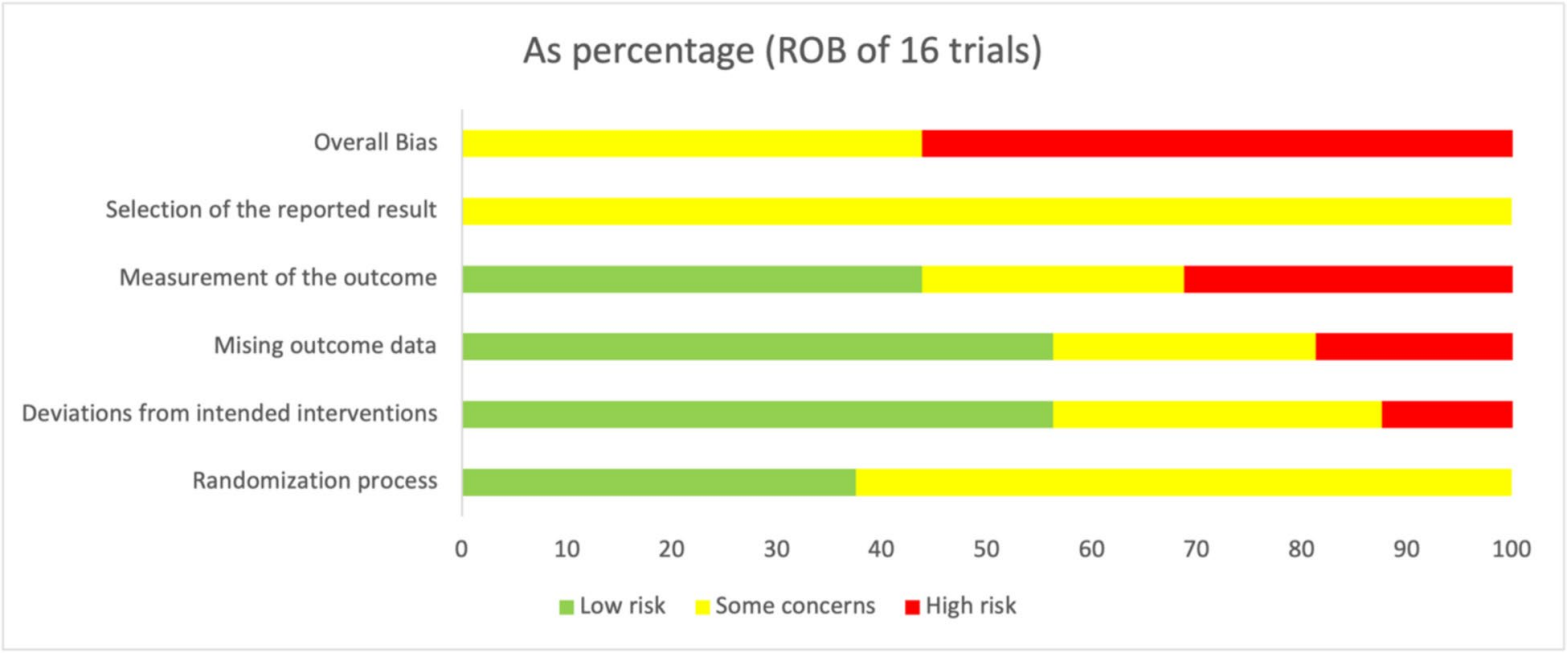
Overall risk of bias assessment as percentages

The results of the risk-of-bias assessment were visually represented in two key formats within the clinical trials section. Individual graphical representations were created for each trial, showcasing the risk-of-bias judgments for all five domains (Fig. 5). Additionally, a summarized bar chart (Fig. 6) was generated to illustrate the distribution of risk-of-bias judgments across all included studies, categorized into “low risk,” “some concerns,” and “high risk” for each domain and overall bias.

Sixteen RCTs were assessed, and the findings highlighted varying bias levels across the different domains, as shown in this table (Table 4). Regarding the randomization process, 37.5% of the studies were rated as low risk, while 62.5% exhibited some concerns. For deviations from intended interventions, 56.3% of studies were rated as low risk, 31.3% as having some concerns, and 12.5% as high risk. Regarding missing outcome data, 56.3% of the studies were considered low risk, 25% had some concerns, and 18.8% were judged to have high risk. Bias in the measurement of outcomes showed 43.8% of studies with low risk, 25% with some concerns, and 31.3% with high risk. Bias in the selection of reported results was a domain where all studies (100%) exhibited some concerns, with no studies rated as low or high risk. Finally, when considering the overall risk of bias, none of the studies were categorized as low risk, with 43.8% having some concerns and 56.3% as high risk.

**Table 4:** Summary of risk of bias assessment

Total number of studies = 16	Randomization process	Deviations from intended interventions	Missing outcome data	Measurement of the outcome	Selection of the reported result	Overall bias
Low risk	37.5	56.3	56.3	43.8	0	0
Some concerns	62.5	31.3	25	25	100	43.8
High risk	0	12.5	18.8	31.3	0	56.3

These findings stress the prevalence of methodological concerns in the reviewed studies, particularly regarding the selection of reported results and the overall risk of bias. The graphical and tabular summaries presented in Figs. 5 and 6 provide a comprehensive visualization of these results, offering valuable insights into the methodological rigor of the included studies.

#### Qualitative data synthesis

A thorough qualitative synthesis was implemented to narratively integrate the findings from the included studies and provide a structured approach to inspect the evidence by organizing the results around predetermined thematic areas. This ensured a clear and coherent understanding of the efficacy and safety of phytotherapy for hair loss. The first key area of the synthesis focused on the effectiveness and safety of phytotherapy across various forms of hair loss. This evaluation included studies exploring subjective and objective outcomes related to hair regrowth, density, thickness, and patient-reported satisfaction with treatment. Safety outcomes were also highlighted, with an emphasis on adverse events and tolerability associated with the use of herbal interventions.

The synthesis also included comparative analyses of phytotherapy against standard treatments, such as different minoxidil solution concentrations (2%, 3%, 5%) widely used in managing hair loss. These comparisons provided insight into whether phytotherapy offers comparable or superior efficacy and safety profiles and whether it could be an alternative or adjunct to conventional treatments. Additionally, subgroup analyses were performed based on the underlying causes of hair loss, such as genetic (AGA), immune-mediated (AA), hormonal (hypothyroidism or PCOS), or chemotherapy-induced hair loss. These subgroup analyses helped to elucidate whether specific phytotherapeutic agents demonstrated differential efficacy or safety in addressing distinctive reasons for hair loss.

The proposed mechanisms of action of phytotherapy were also synthesized to provide a deeper understanding of how plant-based therapies may promote hair regrowth. Mechanisms such as anti-inflammatory properties, hormonal modulation, antioxidant effects, and improved scalp microcirculation were explored, along with evidence from in vivo and in vitro studies supporting these pathways. This systematic evaluation also helped to identify gaps in the current evidence, particularly in translating in vitro findings into clinical outcomes, highlighting areas where further research is demanded.

The risk of bias and quality of evidence across the included studies were critically appraised as part of the synthesis. This evaluation provided a framework for the reliability and generalizability of the findings, linking the methodological rigor of studies to the strength of their conclusions. Visual evidence of hair loss changes, such as before-and-after images and scalp microscopy findings, were included when available to support the narrative synthesis. These visual representations and a summary of findings were synthesized into a clear tabulated format, aiding in outcomes interpretation.

The synthesis ended by discussing the limitations of the current body of evidence, such as short follow-up periods, small sample sizes, and variability in intervention details, which limit the ability to draw definitive conclusions. Recommendations for future research emphasized the need for larger, well-designed, randomized controlled trials with standardized outcome measures, longer follow-up durations, and exploration of phytotherapy’s role in combination regimens. Additionally, the need for mechanistic studies to bridge preclinical and clinical evidence was highlighted, along with addressing the methodological inadequacies that may have contributed to bias in the existing studies. This qualitative synthesis serves as a foundation for advancing research and clinical practice in the phytotherapy specialty for hair loss.

#### Hair loss due to genetic factors (androgenic alopecia)

AGA, previously mentioned as male or female pattern baldness, is a prevalent condition characterized by progressive hair loss. It affects a significant proportion of the population, with estimates suggesting up to 80% of men and 50% of women will experience some degree of AGA in their lifetime (Cheyasak, et al. 2024). The underlying mechanisms of AGA involve a complex interplay of genetic predisposition, hormonal influences, and inflammatory processes (Pekmezci et al. 2018). The key player is DHT, a potent androgen converted from testosterone by the enzyme 5α-reductase. DHT binds to androgen receptors in hair follicles, leading to follicles’ miniaturization, shortening of the anagen (growth) phase, and, ultimately, hair thinning (Akhbari, et al. 2024). Conventional therapies for AGA, such as topical minoxidil and oral finasteride, often come with undesirable adverse effects (Tanuphol, et al. 2024). This has fueled the search for alternative treatments, like using medicinal plants, and gained considerable interest. Research into the potential of herbal medicine for managing AGA has yielded promising results, suggesting certain plant extracts possess antiandrogenic, anti-inflammatory, and antioxidant properties that can modulate the hair growth cycle and promote regeneration (Lueangarun and Panchaprateep 2020). However, the evidence base remains somewhat limited, with a need for more rigorous clinical trials to confirm the efficacy and safety of these natural interventions.

Several clinical trials have explored the effectiveness of various phytotherapeutic interventions for AGA. For instance, a triple-blind, randomized, controlled trial investigated the effects of oral Amla (*Phyllanthus emblica* L.) syrup on female androgenetic alopecia (FAGA). This study, conducted by Akhbari, et al. (2024), involved 60 women aged 18 to 60, with 27 completing the intervention (10 mL of Amla syrup taken thrice daily) and 25 completing the placebo arm for 3 months. The results demonstrated a significant increase in the anagen-to-telogen ratio in the amla group compared to the placebo group (*P* = 0.002) using TrichoScan for assessment without a significant improvement in the other hair growth parameters like the terminal-to-vellus hair ratio, hair count, and hair thickness (*P* > 0.05). Additionally, both physician and patient satisfaction scores were higher in the amla group at 12 weeks of intervention (*P* < 0.001). The formula had no remarkable side effects. Only one case of mild constipation was reported in one of the participants after 1 month of consuming amla syrup. Moreover, another randomized, comparative trial examined the efficacy of a commonly known ingredient in hair care products, which is rosemary oil (*Rosmarinus officinalis* L.) versus 2% topical minoxidil, in the treatment of AGA in 100 male patients. Fifty of them were randomly assigned to use 2 mL of rosemary oil for 6 months on the frontoparietal and vertex area of the scalp with gentle massage. Apparently, the findings of Panahi et al. (2015) indicated that both treatments significantly increased hair count after 3 and 6 months of use (*P* < 0.05) and that rosemary oil was as effective as 2% minoxidil in the treatment of AGA. In other words, there was a significant improvement in hair loss within groups but not between groups (*P* > 0.05), as well as a higher frequency of scalp itchiness within both groups (*P* < 0.05). However, scalp itchiness was more evident in the minoxidil group compared to the rosemary one at both assessed endpoints of the trial (*P* < 0.05), suggesting utilizing rosemary as a safer alternative to the minoxidil solution.

Another methodology followed in the trials is to adopt certain extraction procedures for the herbal plants; for example, a double-blind, randomized, placebo-controlled study assessed the effectiveness and safety of a hair tonic containing 1% teak (*Tectona grandis* L.) leaf extracted through cold ethanol maceration. (Tanuphol, et al. 2024) included 81 male subjects with AGA assigned to 3 arms of 27 using teak hair tonic (HT-teak), 5% minoxidil, or placebo for 24 weeks. The teak leaf extract demonstrated promising results in increasing target area hair count (TAHC) at week 12 (mean ± SE, 106.45 ± 2.32; *P* = 0.023) without having further statistical significance until week 24. Additionally, 48.15% of HT-teak subjects reported reducing hair shedding at weeks 4 and 16 compared to placebo (*P* = 0.041, 0.008, respectively). Both the HT-teak and minoxidil groups exhibited a significant increase in the anagen-to-telogen ratio (A/T) at week 24 (mean ± SE, 139.22 ± 10.23; *P* = 0.002) and week 20 (mean ± SE, 135.89 ± 11.51; *P* = 0.029), respectively, while the placebo group showed no observable signs of hair regrowth. The HT-teak group reported the highest satisfaction scores on overall hair changes (mean ± SE, 2.04 ± 0.71; *P* = 0.037). There were no indications of skin irritation or systemic effects on sexual dysfunction and palpitation after the 6 months of HT-teak application, unlike minoxidil, whose subjects reported scalp dryness and itching, posing its potential as an effective and safe hair growth promoter in AGA. Moreover, a unique extraction method was performed by Jae-Suk et al. (2015) in a double-blind, randomized, controlled trial to prepare the rice bran supercritical CO2 extract (RB-SCE) formulation. Rice bran (*Oryza sativa* L. var. *japonica*) was evaluated for its effects by dermal application of 0.5% RB-SCE in 21 alopecia patients (9 male, 12 female) out of 43 enrolled in the RCT. The findings revealed that RB-SCE significantly increased hair density after 16 weeks in the men using it compared to placebo (*P* = 0.034). RB-SCE also significantly increased hair diameter by about 17 μm from baseline (*P* < 0.05) after the 4 months in both male and female participants, which was 3.1-fold greater than the placebo group (*P* = 0.001). Furthermore, the expert panel observed improved hair growth in males in the RB-SCE group after 16 weeks of treatment (score = 0.89 ± 0.601; *P* = 0.002) while no improvement in hair growth was observed in the placebo group (0.08 ± 0.862, *P* = 0.753). The overall satisfaction of the RB-SCE group was also significantly higher than that of the placebo group in both males and females at 16 weeks (*P* = 0.005). No adverse reactions such as itching, prickling, burning, stinging, stiffness, tightness, burning of the eyes, weeping, erythema, edema, scaling, papule, or any other reaction related to RB-SCE were reported, showing its prospect to be a safe and effective therapy for AGA.

On the other hand, various trials used combinations and mixtures of herbs to offer a multi-faceted solution to hair loss due to AGA. One randomized, double-blind, controlled trial was conducted by Cheyasak, et al. (2024) to compare the efficacy and safety of an herbal extract combination solution called Redenyl®, containing dihydro quercetin glucoside, epigallocatechin gallate glucoside, zinc, and glycine, to 3% minoxidil solution in 60 participants (30 males and 30 females). Both groups experienced significant increases in total hair count, measured in a 1-cm^2^ area on the mid-scalp using dermoscopy (herbal: mean ± SD = 345.03 ± 119.26; *P* < 0.001; minoxidil, 391.50 ± 183; *P* < 0.001), and Hair Mass Index (HMI), measured using a Haircheck system® in a 2 × 2 cm^2^ area (herbal = 25.83 ± 171.8; *P* = 0.001, minoxidil = 33.7 ± 15.17; *P* < 0.001) by the end of the 6 months. No statistically significant differences in efficacy were observed between the two groups for total hair count and HMI at week 24 (*P* = 0.250, *P* = 0.065). Additionally, no local adverse effects were observed in both groups regarding both sexes, concluding that Redenyl® is equivalent to the 3% minoxidil solution.

Another proprietary blend of natural ingredients consisting of standardized botanicals, vitamins, and minerals, known as ALRV5XR, was tested by Feldman et al. (2022) in a double-blind, placebo-controlled trial. This study assessed the impact of ALRV5XR on hair regeneration in 37 subjects with AGA (20 women and 17 men) of 77 patients joined for 6 months. ALRV5XR was administered as an oral capsule of 842 mg twice daily and applied topically as a shampoo (3–7 mL daily), conditioner (3–7 mL daily), and follicle serum (1 mL daily). It demonstrated significant increases in terminal hair density, measured using Phototrichogram, in both men and women, with women experiencing a 30.1 THs/cm^2^ increase (*P* = 0.0002) and men a 21.0 THs/cm^2^ increase (*P* = 0.0014) after 24 weeks. Additionally, the terminal-to-vellus hair ratio increased significantly in men only by 33% (*P* = 0.0033) in contrast to a significant increase in growth rate in women only by 10% (30.7 μm/24 h; *P* < 0.0001). The hair shedding rate also declined in men only by 21.5% (*P* = 0.0158) as opposed to a non-statistically significant decrease trend in women (21.8%; *P* > 0.05). The safety profile was evident because no clinically relevant adverse signals or adverse events were reported, leading to proving its role in the improvement of hair growth, density, and thickness by reactivating dormant hair follicles and thickening miniaturized hairs.

Lueangarun and Panchaprateep 2020) also investigated the efficacy of an herbal extract combination composed of acetyl tetrapeptide-3, biochanin A (*Red clover* extracts), *Panax Ginseng* extract, and *Salvia officinalis* oil. The conducted triple-blind, randomized, controlled trial compared this mixture to 3% minoxidil lotion in 32 individuals with mild to moderate AGA (16 men with Hamilton-Norwood Stage III-IV vertex male pattern hair loss [MPHL]; 16 women with Ludwig Type I-II, female pattern hair loss [FPHL]). These patients were randomly assigned to use either the herbal combination formula, known as Hirsuit™ hair tonic (17 subjects), or 3% minoxidil lotion (15 subjects), both applied as 1 mL twice daily to the vertex area of the scalp for 24 weeks. The study found no significant difference in clinical efficacy between the two groups (hair count: *P* = 0.370 at week 12, *P* = 0.306 at week 24; HMI: *P* = 0.158 at week 24), with both showing improvements in terminal hair counts (herbal: mean ± SD = 110.2 ± 28.7; *P* = 0.009; minoxidil, 122.1 ± 29.07; *P* = 0.002) and HMI (herbal, 45.9 ± 26.3; *P* = 0.008; minoxidil, 61.1 ± 31.5; *P* = 0.026) after 24 weeks. The expert panel photographic assessment observed response to both treatments in most patients at 24 weeks, with no statistically significant difference in an increase of terminal hair counts (8.3%; *P* = 0.009 and 8.7%; *P* = 0.002 at 24 weeks in the herbal extract combinations and the 3% minoxidil solution groups, respectively). Additionally, there was no statistically significant difference in patient satisfaction between the groups at week 24 regarding hair density (herbal 68.75% vs. minoxidil 76.92%; *P* = 0.85) and hair thickness (herbal 78.92% vs. minoxidil 69.23%; *P* = 0.92). Notably, the herbal extract combination demonstrated a better safety profile, as one participant in the minoxidil group experienced scalp eczema after using it for 6 weeks, leading to her switching to the herbal group, which is proven as an alternative treatment for AGA.

Another newly registered combination, with antiandrogenic, anti-inflammatory, and antioxidative properties, was tested in a single-blind, placebo-controlled trial by Pekmezci et al. (2018). This trial evaluated the efficacy of applying a proprietary herbal shampoo and solution containing six different herbal extracts (*Urtica urens* leaf extract, *Urtica dioica* root extract, *Matricaria chamomilla* flower extract, *Achillea millefolium* aerial part extract, *Ceratonia siliqua* fruit extract, *Equisetum arvense* leaf extract) in 120 subjects (60 males, 60 females) with AGA (54) or TE (66). The topical application instructions were as follows: use 5 ml of herbal extracts shampoo on wet hair three times a week and leave to foam for 3–4 min before rinsing with 3 ml of the same herbal extract solution on dry hair twice daily, massage into the scalp and leave for at least 4–6 h. The study demonstrated that the herbal formulations were more effective in preventing and reducing hair loss compared to the placebo at every assessment point (done every month for 6 months; hair loss decreased by about 92% at 6 th month, *P* < 0.000001) using the pull test, which is the number of hairs extracted during gentle traction. The Phototrichogram was also used to measure hair count in a targeted area of 1.8 cm^2^, showing the greatest number of hair count (225) in the third group using both herbal shampoo and solution. Additionally, anagen/telogen ratios improved significantly in this group using both herbal formulations, whereas telogen hair was decreased by about 20% and anagen hair increased by 30% from baseline. Consequently, the concomitant use of the shampoo and solution was found to be more effective than single-product use, as evident from the results as well as the patient self-assessment survey (*P* < 0.000001).

The only RCT that evaluated the synergism of using herbal formulation with conventional minoxidil therapy in AGA was held by Masoud et al. (2020). This randomized, double-blind, controlled trial investigated the effectiveness of a topical herbal solution (THS) containing extracts of *Rosmarinus officinalis* (rosemary oil), *Olea europaea* (olive oil), and *Serenoa repens* (saw palmetto) and 5% minoxidil topical solution (MTS) compared to MTS alone in 24 men with mild to moderate AGA (type III-V on the Hamilton-Norwood classification) for 9 months. The MTS + THS group was significantly superior to the MTS group after 36 weeks of therapy in the hair diameter improvement measured using a digital micrometer (62.67 ± 7.19, 50.58 ± 7.38, respectively; *P* = 0.001). On the contrary, the hair density of the MTS + THS group significantly increased within the group (96.92 ± 3.06 hairs/cm^2^; *P* = 0.003); however, it was not statistically different when compared to the MTS group (*P* = 0.153) at week 36 after using both formulations twice daily (2 mL/day morning and evening). Additionally, the MTS + THS group was superior to the MTS group in the patients’ self-assessment questionnaire, measuring the quality of therapy after 36 weeks of use in terms of increasing hair growth, reducing hair loss, and improving hair appearance (*P* < 0.05). The findings supported using the combination as it had significant positive effects on AGA patients than the minoxidil alone.

These aforementioned studies have investigated the mechanisms of action of plant-based treatments for AGA, revealing multiple pathways through which these natural compounds may be effective. Many of these treatments appear to target the 5α-reductase enzyme, which converts testosterone to DHT, the key hormone in AGA development. For example, *Phyllanthus emblica* (Amla) syrup, *Rosmarinus officinalis*, *Tectona grandis* (teak leaf extract), *Urtica dioica* and *Ceratonia siliqua*, and *Serenoa repens* have all demonstrated 5α-reductase inhibitory activity. In addition to enzyme inhibition, stimulation of dermal papilla cells (DPCs) is a common mechanism. *Phyllanthus emblica* and a combination of dihydroquercetin-glucoside (DHQG), epigallocatechin gallate glucoside (EGCG), glycine, and zinc have been shown to enhance DPC proliferation. Biochanin A, acetyl tetrapeptide-3, and ginseng extracts also promote DPC activity by stimulating the dermal papilla extracellular matrix (ECM). Furthermore, EGCG was found to lengthen hair follicles ex vivo and minimize the detrimental effects of DHT. Moreover, many plant-based treatments for AGA exert anti-inflammatory and antioxidant effects, like those using *Phyllanthus emblica*, *Urtica urens*, *Achillea millefolium*, and *Equisetum arvense*, which may help reduce the inflammation and oxidative stress involved in AGA. These mechanisms, combined with others, such as improved blood circulation to the scalp and promotion of wound healing, contribute to the overall effectiveness of these herbal therapies for AGA.

While these trials provide encouraging evidence for the potential of phytotherapy in managing AGA, several limitations warrant consideration that need to be addressed in future research. A common issue is the small sample sizes used in trials, including those by Akhbari, et al. (2024); Masoud et al. 2020), and (Tanuphol, et al. 2024), which may limit the generalizability of findings to broader populations. Some studies also have short follow-up periods (e.g., 12 weeks in Akhbari, et al. (2024)), which are insufficient for assessing the long-term efficacy and safety of the interventions. Additionally, studies often use varied outcome measures like hair counts, HMI, and anagen-to-telogen ratios, making it difficult to compare results across trials. The lack of placebo groups in some studies also makes it hard to attribute the benefits to the treatment rather than the placebo effect. Furthermore, some studies, such as the one by Tanuphol, et al. (2024), do not record comprehensive patient history data, such as genetic history or hormonal levels, that could affect treatment outcomes. Finally, variations in application methods, such as topical solutions, shampoos, and oral treatments, add to the challenge of comparing and generalizing results. Therefore, future research should include larger, well-designed, randomized, controlled clinical trials with longer follow-up periods, standardized outcome measures, comprehensive patient data, and evaluations of long-term safety and impact on quality of life in individuals with AGA, including its psychological and social implications. By addressing these research gaps, we can gain a more comprehensive understanding of the potential role of phytotherapy as a safe and effective treatment option for individuals seeking to manage and potentially reverse the effects of androgenic alopecia.

#### Hair loss due to stress (telogen effluvium)

TE is a common type of hair loss characterized by excessive hair shedding from the scalp. It typically occurs when a significant number of hair follicles prematurely enter the telogen (resting) phase of the hair growth cycle. This shift in the hair cycle can be triggered by various factors, including stress, hormonal changes, nutritional deficiencies, certain medications, and medical conditions. While TE is generally a temporary condition, it can be distressing for those experiencing it, as it often leads to noticeable hair thinning (Pekmezci et al. 2018). Conventional management of TE usually focuses on addressing the underlying cause and reassuring the patient that hair growth will eventually resume. However, alternative therapies may be sought in some cases, particularly when the cause is unclear or difficult to address. Phytotherapy, with its potential to modulate hair growth and reduce inflammation, has emerged as an intriguing option for TE management (Pekmezci et al. 2018). The following clinical trials offer insights into the benefits of using phytotherapy for managing TE.

As discussed in the AGA section, (Feldman et al. 2022) conducted that double-blinded controlled trial to investigate the efficacy of ALRV5XR, the botanical blend, in treating both AGA and TE. This trial enrolled 92 subjects, of whom 23 women had a secondary diagnosis of TE, and results showed that ALRV5XR significantly increased terminal hair density in women with TE, similar to the efficacy observed in the overall cohort of women. This suggests that ALRV5XR may be effective in promoting hair regrowth in individuals with TE, regardless of whether it is a primary or secondary diagnosis. Besides, the safety profile, proven by unreported adverse events in the trial, supports its use. On the other hand, the single-blind trial of Pekmezci et al. (2018) assessed the efficacy of the herbal extracts-containing shampoo and solution in 120 subjects, 66 diagnosed with TE. The study found that these herbal formulations were significantly more effective than placebo in preventing and reducing hair loss at all assessment points (every month for 6 months), as elaborated in the AGA section. For example, the pull test, which measures hair shedding, showed a mean decrease in hair loss ranging from 28.3 to 49.1% in the herbal shampoo and solution groups compared to 12.3% in the placebo group in the first month. Additionally, the anagen/telogen ratio, a measure of the proportion of growing versus resting hair follicles, significantly improved in the group using the herbal formulations in both diagnoses (*P* < 0.05). It was also proven that using both the shampoo and solution together provided synergistic benefits compared to using either product alone in TE as found in AGA patients.

The (Feldman et al. 2022) and (Pekmezci et al. 2018) studies suggest that plant-based treatments for TE work through multiple mechanisms. The ALRV5XR treatment in the (Feldman et al. 2022) study targets hair follicle stem cell activation, promotes healthy hair growth, modulates growth factors, and influences hormone mediators to reactivate dormant telogen follicles, especially in women, while also thickening miniaturized hair in men. Besides, the herbal extracts in the (Pekmezci et al. 2018) study utilize antiandrogenic effects, mainly through *Urtica dioica*, which inhibits 5α-reductase, reducing DHT levels, as explained in the AGA section. These extracts also exhibit anti-inflammatory and antioxidative properties through components like polysaccharides, caffeic malic acid, and flavonoids from *Urtica urens*, *Equisetum arvense*, *Achillea millefolium*, *Matricaria chamomilla*, and *Ceratonia siliqua*, which reduce inflammation and oxidative stress. These herbs also include compounds that promote angiogenesis, improve scalp blood flow, and provide essential vitamins, minerals, and trace elements that are vital for hair follicle health.

While these trials offer encouraging evidence for the potential of phytotherapy in managing TE, further research is needed to confirm these findings and address existing limitations. As stated in the AGA section, (Feldman et al. 2022) and (Pekmezci et al. 2018) suffer from small sample sizes, short follow-up periods, and inconsistent outcome measures. Additionally, incomplete patient data, including TE duration and social and medical history, further hinders the ability to assess individual responses to treatment. Moreover, future research should investigate the optimal dosage, frequency, and duration of treatment for different phytotherapy interventions in TE. Besides, both studies included both AGA and TE participants, which makes it hard to differentiate the specific treatment effects for each condition. Since AGA and TE have distinct underlying mechanisms, results from mixed groups may not accurately reflect the effectiveness of each condition. Finally, research should explore the mechanisms of action of plant extracts on the hair growth cycle and evaluate their impact on the quality of life for individuals with TE while ensuring long-term safety monitoring. So, we can gain a more comprehensive understanding of the potential role of phytotherapy as a safe and effective treatment option for individuals seeking to manage telogen effluvium.

#### Hair loss due to immunological factors (alopecia areata)

AA is a common autoimmune disease that causes patchy, non-scarring hair loss on the scalp and potentially other body areas (Kim et al. 2020). While various treatments are available, including corticosteroids and immunosuppressants, none are universally effective or preventive. Consequently, there is growing interest in exploring alternative therapies, such as herbal therapy, for managing AA (Moosavi et al. 2020). Unfortunately, there are not plenty of randomized controlled trials held to investigate the efficacy and safety of plant-based extracts in treating AA. For instance, one study tested a formulation of an immunomodulatory mechanism of action, and another one evaluated AA patients.

The therapeutic effect of a 2% *Urginea maritima* (squill) bulb extract lotion was compared to 0.05% clobetasol propionate lotion in 42 patients diagnosed with AA and with at least 25% scalp hair loss due to AA. Both groups received their assigned treatment twice daily (morning and night being rubbed on the alopecia patches) for 12 weeks in this randomized, double-blinded clinical trial conducted by Moosavi et al. (2020). The primary outcome measure was the regrowth score (RGS) (a semi-quantitative assessment of terminal hair regrowth), patches’ size (using 1 × 1 cm^2^ schablone to be a standardized template), and total number of grown hair (count using lens). While no significant differences in RGS were observed between the two groups after one month of treatment (*P* = 0.359), the squill group showed a significantly greater increase in the proportion of patches with RGS4 (growth of > 75%) at the end of the eighth week (*P* = 0.000). However, by the third month, both groups demonstrated significant improvements in overall RGS, with a statistically significant difference between them for the squill group being ahead in RGS4 (squill 23.3% vs. clobetasol 9.6%; *P* = 0.040). Additionally, both mean hair growth rate and vellus hair growth rate in alopecia patches increased significantly within each group at every evaluation point (every month), yet no significant difference was demonstrated after the third month between the two groups (*P* > 0.05). Furthermore, there was no statistically significant difference between the squill and clobetasol groups in terms of the affected area mean size decrease and patient treatment satisfaction, though side effects reported in the clobetasol group (mild irritation, 40%) were greater than that of the squill group (itching 10%, burning sensation 20%). So, squill extract was deemed effective and safe, especially in promoting hair growth in AA patchy areas.

While (Kim et al. 2020) did not explicitly state that the reason for hair loss in their study was alopecia areata, their findings on the mechanism of action indirectly support the potential of *Centipeda minima* (CMX) in managing hair loss associated with immune dysregulation, which is a hallmark of alopecia areata. The study inclusion criteria focused on patients with “mild to moderate vertex balding,” which could be caused by various factors, including androgenetic alopecia, telogen effluvium, or alopecia areata. However, the researchers conducted a network pharmacological analysis focusing on brevilin A, the active compound in CMX. This analysis revealed that brevilin A modulates the JAK-STAT signaling pathway, which plays a crucial role in the immune response and whose dysregulation has been implicated in the development of alopecia areata. Therefore, although the study does not definitively focus on alopecia areata, the network analysis provides valuable insights into how CMX might exert its effects, potentially explaining the observed hair growth improvements by targeting immune-related pathways. This information could be beneficial in understanding the potential application of CMX in managing alopecia areata. The study by Kim et al. (2020) examined the effects of the once-daily emulsion extract of brevilin A on 72 patients with mild to moderate vertex balding for 24 weeks and measured total hair count, terminal hair count, and anagen hair count. Significant differences in all three hair count measures were observed between the CMX and placebo groups (*P* < 0.001). The CMX group also demonstrated higher counts for total hair (mean difference of 3.5), terminal hair (3.1), and anagen hair (3.6) compared to the placebo group. Notably, a higher proportion of patients in the CMX group experienced significant improvement in all three hair count categories.

Moosavi et al. 2020) and (Kim et al. 2020) explored different plant-based treatments for alopecia areata with distinct mechanisms. (Moosavi et al. 2020) investigated squill extract (*Urginea maritima*) and suggested its traditional use for hair loss may stem from its ability to cause skin redness, which in turn resumes hair growth, possibly due to its cardiac glycosides like scillaren A, alongside other compounds, although the study did not find a significant advantage over clobetasol. Whereas (Kim et al. 2020) focused on *Centipeda minima* and its active compound, brevilin A, which is a JAK3 inhibitor, their findings indicated that brevilin A’s inhibition of the JAK-STAT signaling pathway is crucial to its hair regrowth effects by disrupting the inflammatory process characterized by the infiltration of CD8αβ + NKG2D + T cells into hair follicles. Further analysis by Kim et al. (2020) revealed that brevilin A also targets pathways related to cytokine-cytokine receptor interactions and the MAPK signaling pathway, suggesting a multi-targeted approach to treating hair loss, with CMX demonstrating a more potent JAK3 inhibition than brevilin A alone. The clinical trial also confirmed these findings, showing that the CMX group had significant improvements in hair counts compared to the placebo group.

The studies by Moosavi et al. (2020) and (Kim et al. 2020) suggest plant-based extracts as potential treatments for AA, but their findings are limited by several factors. Both trials, while indicating promise, used small sample sizes, which hindered the generalizability of the results. Furthermore, the variations in outcome measures and assessment methods between the two studies, with (Moosavi et al. 2020) focusing on a regrowth score and (Kim et al. 2020) on hair counts, make it difficult to directly compare their findings. Additionally, the studies have limited information on the long-term efficacy and safety, with follow-up periods of 12 and 24 weeks, respectively, which are insufficient for a chronic condition like AA. Thus, future research should prioritize larger-scale trials with standardized outcome measures designed for AA, as well as longer follow-up periods, and should further elucidate the specific mechanisms of action of plant-based extracts. Exploring phytotherapy’s potential in managing AA represents a valuable avenue for improving treatment options and patient outcomes.

#### Hair loss due to hormonal imbalances

Hair loss, a common symptom associated with hormonal imbalances in endocrine conditions like hypothyroidism and polycystic ovary syndrome (PCOS), can significantly impact individuals’ quality of life and psychological well-being (Ashraf et al. 2022). Hypothyroidism is characterized by the thyroid gland not producing enough thyroid hormones, which can lead to symptoms such as fatigue, cold intolerance, weight gain, and hair loss. PCOS is also an endocrine disorder that can cause an imbalance in sex hormones, leading to symptoms like acne, thinning hair, weight gain, irregular periods, and infertility. While conventional treatments for these endocrine disorders address the underlying hormonal imbalances, they may not fully resolve hair loss. This has led to growing interest in exploring complementary and alternative therapies, such as herbal interventions, to manage hair loss associated with these conditions (Mirgaloybayat et al. 2024). Two recent randomized controlled trials have investigated the effects of plant-based extracts on hair loss in patients with hypothyroidism and PCOS.

The first study, a single-center, double-blinded, placebo-controlled, randomized clinical trial conducted by Ashraf et al. (2022), examined the efficacy of ginger supplementation in relieving persistent hypothyroid symptoms, including hair loss, in patients with controlled primary hypothyroidism. In this double-blind, placebo-controlled trial, 53 hypothyroid patients aged 20–60 years with normal serum TSH concentrations were randomized to receive either 500 mg of ginger twice daily or a placebo for 30 days. While the study focused on a broader range of hypothyroid symptoms, it specifically assessed hair loss as a secondary outcome using the Thyroid Symptom Rating Questionnaire (ThySRQ). It found no significant improvement in hair loss in the ginger group compared to the placebo group (*P* > 0.05) after 1 month, in contrast to the other domains of weight gain, cold intolerance, constipation, memory disturbance, and dry skin, showing a significant difference upon using 1 g of ginger powder daily. This suggests that ginger supplementation, at the dosage and duration used in this trial, may not be effective in managing hair loss associated with hypothyroidism.

The second study by Mirgaloybayat et al. (2024) compared the effects of fenugreek and metformin on clinical and metabolic parameters in women with PCOS, including moderate to severe hair loss. This randomized triple-blinded parallel clinical trial involved 110 women aged 16–40 years with PCOS who were assigned to receive either 333 mg of fenugreek three times daily or 500 mg of metformin three times daily for 2 months. It assessed hair loss as one of the clinical symptoms in the primary outcomes, though the specific assessment method was not explicitly stated, and concluded that both fenugreek and metformin significantly reduced hair loss in patients with PCOS. In the fenugreek group, the McNemar odds ratio was 9.33 (*P* < 0.001), while in the metformin group, the McNemar odds ratio was 3.2 (*P* = 0.027) after 60 days. These findings suggest that both fenugreek and metformin may be beneficial in managing hair loss associated with PCOS.

The studies by Ashraf et al. (2022) and (Mirgaloybayat et al. 2024) offer different insights into the management of hair loss related to hormonal imbalances, with a focus on ginger and fenugreek, respectively. While (Ashraf et al. 2022) found that ginger supplementation did not significantly improve hair loss in hypothyroid patients despite improvements in other symptoms, they suggest that ginger’s mechanism of action is likely related to reducing inflammation and regulating lipid and glucose metabolism, factors that are common comorbidities in hypothyroidism. However, these mechanisms did not translate to a noticeable effect on hair loss in their study population. On the contrary, (Mirgaloybayat et al. 2024) found that fenugreek improved hair loss in women with PCOS, attributing this effect to several mechanisms, primarily fenugreek’s impact on lipid profiles, insulin sensitivity, and hormonal regulation. The study suggests that the linoleic acid in fenugreek seed oil may reduce LH levels, which are linked to hormonal hair loss. Furthermore, fenugreek’s hypolipidemic effects, which help to lower levels of LDL and triglycerides while raising HDL levels and its ability to improve insulin resistance may also indirectly contribute to improved hair growth. Additionally, fenugreek’s potential to reduce symptoms linked to hyperandrogenism, such as hair loss, is a key aspect of its suggested mechanism in the context of PCOS. In summary, while ginger may address some metabolic imbalances, it did not show a significant effect on hair loss; fenugreek, on the other hand, is suggested to improve hair loss through a combination of hormonal modulation, improved lipid profiles, increased insulin sensitivity, and reduced hyperandrogenism, making it potentially beneficial in managing hair loss associated with PCOS.

The contrasting results regarding hair loss between the (Ashraf et al. 2022) and (Mirgaloybayat et al. 2024) trials highlight the need for more targeted research, as hair loss was not a primary outcome in either study, which introduces significant limitations. Both trials utilized relatively short intervention periods, with (Ashraf et al. 2022) using a 30-day period for ginger supplementation in hypothyroid patients and (Mirgaloybayat et al. 2024) using a 60-day period for fenugreek in women with PCOS, which may not be sufficient to observe meaningful changes in hair regrowth. Furthermore, neither study employed standardized, validated tools specifically designed to assess hair loss. (Ashraf et al. 2022) relied on the Thyroid Symptom Rating Questionnaire (TySRQ), which included a question about hair loss but was not designed to measure specific changes in hair growth or density. Similarly, (Mirgaloybayat et al. 2024) used the modified Ferriman-Gallwey scoring method to assess hirsutism, which does not focus on scalp hair loss. This lack of specific assessment tools makes it challenging to draw firm conclusions about the effectiveness of these treatments on hair regrowth. Additionally, both studies had a secondary focus on hair loss, with (Ashraf et al. 2022) primarily focusing on overall hypothyroid symptoms and (Mirgaloybayat et al. 2024) focusing on PCOS symptoms and metabolic markers. This secondary focus may have led to less detailed collection and analysis of data directly relevant to hair loss. Specifically, (Ashraf et al. 2022) did not find a significant improvement in hair loss with ginger supplementation, while (Mirgaloybayat et al. 2024) reported a significant improvement in hair loss with fenugreek but as a secondary finding. By tackling these limitations, future research can provide more robust evidence for the potential role of phytotherapy in managing hair loss associated with hypothyroidism, PCOS, and other endocrine disorders.

#### Hair loss due to medications (chemotherapy-induced)

Chemotherapy-induced hair loss, usually known as chemotherapy-induced alopecia (CIA), is a common and distressing side effect of cancer treatment, often leading to psychological distress and decreased quality of life for patients (Yu et al. 2019). While scalp cooling has been suggested as a potential treatment for CIA, it does not address other chemotherapy-induced side effects and may even exacerbate alopecia in some cases (Yu et al. 2019). Therefore, research continues to explore alternative therapies, including phytotherapy, which is the use of plant-based medicines to manage CIA and other chemotherapy side effects. Recently, two randomized controlled trials have investigated the efficacy and safety of specific Chinese herbal medicines in managing chemotherapy-induced side effects, including alopecia, in breast cancer patients. Additionally, a newly registered trial, held in CHUV Lausanne Switzerland, on ClinicalTrials.gov at the NIH (ID NCT02919735) is investigating the CG 428 cutaneous solution efficacy and safety in preventing CIA, though the results are not yet available. This ongoing research highlights the continued interest in finding effective treatments for this distressing side effect.

The first RCT, conducted by Yu et al. (2019), examined the effects of Xiaoaiping, a drug primarily composed of the Chinese herb *Marsdeniae tenacissimae*, on 93 adult patients with breast cancer who had undergone radical mastectomy. Patients were randomly assigned to either a control group receiving routine chemotherapy alone (a total of 47 patients), or a combined group receiving routine chemotherapy and Xiaoaiping (a total of 46 patients). Furthermore, the intervention group had routine chemotherapy consisting of six cycles of Docetaxel (75 mg/m^2^) and Adriamycin (50 mg/m^2^) every 21 days together with 7.2 g/day of Xiaoaiping three times daily for 2 weeks during each chemotherapy cycle. The severity of alopecia was assessed using the WHO criteria, which categorizes alopecia into grades from 0 (no hair loss) to 4 (complete and irreversible alopecia). The researchers have found significantly fewer patients with severe alopecia (grades 3–4) in the combined group compared to the control group (15.2% vs. 34%, respectively; *P* < 0.001) compared with the mild to moderate alopecia (grades 1–2) which was fewer in the control group by 18.8%. Additionally, according to the Kaplan–Meier curve, the combined group possessed a longer alopecia-free time, defined as the duration from the study’s start to the first observation of alopecia. Beyond alopecia, the study also investigated the impact of Xiaoaiping on other common chemotherapy side effects, including nausea and vomiting, diarrhea, decreased white blood cell count, and elevated AST levels. The combined group experienced significantly less severe side effects compared to the control group (*P* < 0.05). Furthermore, patients in the combined group reported a better quality of life, as assessed by the Total Functional Assessment of Cancer Therapy–Breast (FACT-B) score after the treatment, compared to the control group (*P* < 0.05).

The second study investigated the effects of a personalized traditional Chinese medicine (TCM) regimen on 80 patients with HER2-positive breast cancer who had undergone radical mastectomy. Patients enrolled in this (Xu et al. 2021) trial were randomized to receive either chemotherapy alone (3-week regimen of trastuzumab plus paclitaxel) or chemotherapy plus the twice-daily Chinese medicine regimen, which was tailored to each patient’s needs. This TCM is composed of 50 mL liquid of specific tradictional Chinese medicine formula prepared by boiling and containing Poria cocos (Fu Ling) 10 g, atractylodes (Bai Zhu) 10 g, ginger pinellia (Jiang Ban Xia) 10 g, Thunberg fritillary bulb (Zhe Bei Mu) 10 g, radix curcumae aromaticae (Yu Jin) 10 g, Scutellaria (Huang Qin) 10 g, zedoary (E Zhu) 10 g, orange fruit (Zhi Qiao) 10 g, dried tangerine peel (Chen Pi) 10 g, solanum lyrate (Bai Ying) 10 g, chicken’s gizzard-membrane (Ji Nei Jin) 10 g, turtle carapace (Bie Jia) 10 g, licorice root (Gan Cao) 10 g, and centipede (Wu Gong) 10 g. While this study did not explicitly state their method for assessing alopecia, they did note that they measured “symptom dimensions” for various side effects, including hair loss, at the beginning and end of the 6-month treatment period. The researchers observed a significantly greater improvement in hair loss symptoms in the group receiving chemotherapy plus Chinese medicine compared to the chemotherapy alone group (39.02 ± 2.34 vs. 47.44 ± 5.56, respectively; *P* = 0.003).

The studies by Yu et al. (2019) and (Xu et al. 2021) suggest different mechanisms by which traditional Chinese medicine may combat chemotherapy-induced hair loss. Yu et al. (2019) found that Xiaoaiping, derived from *Marsdeniae tenacissimae*, improves alopecia by mitigating the side effects of chemotherapy, potentially through direct anti-tumor effects and reducing chemotherapy toxicity. While the specific mechanism related to hair loss is not detailed, the study implies that Xiaoaiping helps reduce the overall toxic effects of chemotherapy, reducing the severity of alopecia. On the other hand, (Xu et al. 2021) used a personalized TCM approach where the specific herbs used were selected for their properties in managing a range of chemotherapy side effects, including hair loss. While the study did not isolate specific mechanisms for hair loss, it implies that the combination of various herbs, such as *ginger pinellia* for preventing vomiting, *orange fruit* and *dried tangerine peel* for gastrointestinal motility, and *solanum lyrate* for clearing heat and removing toxins, collectively work to reduce the severity of hair loss by improving the body’s overall response to chemotherapy. The (Xu et al. 2021) study suggests that a multi-faceted approach, with herbs working synergistically to address different aspects of chemotherapy toxicity, effectively reduces CIA.

Both studies demonstrate the potential benefits of Chinese herbal medicines in reducing chemotherapy-induced hair loss and related side effects; however, their findings are limited by several factors. The small sample sizes in both studies (93 participants in Yu et al. (2019) and 80 in Xu et al. (2021) limit the generalizability and statistical power of their results. Additionally, the Xu et al. (2021) study lacks detailed methods for assessing alopecia, compromising the reliability of its findings, whereas (Yu et al. 2019) used the WHO criteria for alopecia grading, providing a more standardized approach. Neither study offers long-term efficacy or safety data, with follow-up periods inconsistently reported; (Xu et al. 2021) followed patients during a 6-month chemotherapy period but did not address hair loss beyond this timeframe, while (Yu et al. 2019) measured “alopecia-free time” but did not specify an overall follow-up duration. The personalized herbal treatments by Xu et al. (2021), though tailored to patient needs, introduce variability that hinders result standardization, while (Yu et al. 2019) employed a more uniform intervention. Both studies lacked a detailed exploration of the molecular mechanisms involved; (Yu et al. 2019) linked Xiaoaiping to anti-tumor and toxicity-reducing effects, while (Xu et al. 2021) suggested a combination of multi-action herbs, but neither provided specific pathways. Despite these limitations, the studies highlight the promise of phytotherapy in managing CIA and improving cancer patients’ quality of life, emphasizing the need for more robust, standardized research in this field.

#### Hair loss due to normal aging

Hair loss associated with aging is a common concern that can affect both men and women, often leading to psychological distress and reduced self-esteem. Aging is associated with increased hair shedding and/or hair thinning owing to the hair follicle miniaturization. Oxidative stress and inflammation are also considered contributors to tissue aging, including in skin and hair follicles (Campiche et al. 2022). While various factors contribute to age-related hair loss, including genetics and hormonal changes, the search for effective and safe treatments continues (Somboonwatthanakul et al. 2024). The medicinal plants’ use has emerged as a possible approach to managing age-related hair loss due to their perceived natural and gentle properties. Antioxidants, such as plant extracts rich in phenols and flavonoids, are being investigated as a solution to counteract hair loss (Campiche et al. 2022). Two recent studies have inspected the effects of specific plant extracts on hair loss in individuals experiencing age-related hair thinning.

The first study by Campiche et al. (2022) examined the impact of an extract derived from the alpine plant *Leontopodium alpinum* (Edelweiss) on hair growth. The researchers conducted both ex vivo experiments using human hair follicles from three donors and an in vivo randomized placebo-controlled single-center clinical trial involving 60 healthy human subjects. In the ex vivo experiments, treatment with the 0.001% Edelweiss extract significantly prolonged the anagen (growth) phase of the hair cycle, as evidenced by a greater number of hair follicles remaining in the anagen phase through stimulated hair matrix keratinocyte proliferation, and a decrease in the hair cycle score (*P* < 0.05) which reflects the inhibition of premature progression of hair follicles into the catagen (regression) phase. On the other hand, the in vivo clinical trial involved 60 healthy Caucasian volunteers (both men and women) experiencing hair loss; 30 of them (28 females and 2 males) applied a leave-on serum (ALPAFLOR®) containing 3% Edelweiss extract daily for 150 days. The results showed a statistically significant increase in hair density (about 13,200 new hairs for the whole scalp) measured as the number of hairs per square centimeter and analyzed by TrichoScan® in the group using the Edelweiss formulation compared to the placebo group (+ 22 hair/cm^2^; *P* < 0.001). Additionally, there was a strong trend of an increased anagen-to-catagen/telogen ratio analyzed using Trichogram® after 5 months in the Edelweiss group, suggesting a clinically yet not statistically significant shift towards a more active hair growth phase (*P* = 0.07).

The second trial investigated the effects of an experimental hair tonic formulated with 2% broken riceberry, 1% crude riceberry oil, and 2% extracts from three Thai herbs (*Catunaregam tomentosa*, *Acacia concinna*, and *Tinospora crispa*) on hair loss, grey hair, and dandruff. This randomized, double-blinded, placebo-controlled trial was conducted by Somboonwatthanakul et al. (2024) and involved 20 healthy Thai subjects (predominantly female) aged between 20 and 60 years to test the experimental hair formulation against a placebo and a commercial hair tonic containing *Giffarine Herbita*. Participants were assigned to two groups, each testing two products (experimental vs. placebo and experimental vs. commercial). They applied the hair tonic daily (5 drops to the designated side of the scalp) for 30 days. Both age groups (older and younger) using the experimental hair tonic demonstrated significant reductions in hair loss (70.9 ± 10.5 and 48.0 ± 10.9, respectively; *P* < 0.05) after 1 month. Notably, this experimental hair tonic was deemed more effective in the older group than the younger one in reducing hair loss. On the contrary, this efficacy is reversed regarding grey hair reduction, where the experimental formula is more effective in the younger group than the older one (26.9 ± 10.7 vs. 20.0 ± 1.2, respectively; *P* < 0.05). However, there was no statistically significant difference in hair loss reduction between the experimental hair tonic and the commercial hair tonic within each age group, unlike the difference in the grey hair reduction, in which the experimental formula was superior.

The *Leontopodium alpinum* (Edelweiss) extract, as studied by Campiche et al. (2022), appears to combat age-related hair loss by prolonging the anagen phase through stimulating dermal papilla inductivity, upregulating versican and alkaline phosphatase, which promote the WNT pathway and maintain hair matrix keratinocyte proliferation. In contrast, the hair tonic developed by Somboonwatthanakul et al. (2024) utilizes broken riceberry and crude riceberry oil along with Thai herbal extracts, including *Catunaregam tomentosa*, *Acacia concinna*, and *Tinospora crispa*, to address multiple aspects of hair health. The broken riceberry and crude riceberry oil provide antioxidant activity and stimulate melanogenesis, while the herbal extracts contribute surfactant, cleansing, and conditioning properties, as well as anti-dandruff and anti-balding effects. This combined action of rice by-products and herbal extracts is suggested to manage hair loss through a broader approach, with antioxidant, growth-stimulating, and scalp-nourishing effects. Both approaches offer distinct mechanisms for managing age-related hair loss, with the Edelweiss extract targeting the hair growth cycle and the Thai formula offering a more multi-faceted approach.

While both trials present encouraging results, several specific limitations require consideration. The study by Campiche et al. (2022) utilized a small sample of ex vivo hair follicle donors, with only three donors, potentially limiting the generalizability of the in vitro findings. Furthermore, the clinical trial involved 60 participants, which, while providing some initial evidence, might not be fully representative of the broader population. The study also did not explore potential variations in response to the Edelweiss extract based on individual factors, such as specific causes of hair loss, which could influence the effectiveness of the treatment. Similarly, the study by Somboonwatthanakul et al. (2024) involved only 20 subjects, a sample size that raises concerns about the statistical power and generalizability of the findings. The study also grouped participants with hair loss, grey hair, and dandruff without specifying the exact cause or severity of hair loss due to aging. This makes it challenging to isolate the effects of the hair tonic, specifically on age-related hair loss. Moreover, both studies evaluated the efficacy of the plant extracts over relatively short durations, with the Campiche et al. (2022) (Campiche et al. 2022) study lasting 5 months and the (Somboonwatthanakul et al. 2024) study being conducted over only 30 days. The lack of long-term follow-up in both studies makes it impossible to assess the sustained efficacy and safety of these treatments. Future research should focus on recruiting participants with clearly defined age-related hair loss, utilizing larger sample sizes, targeting longer follow-up periods, and assessing potential confounding factors. These limitations highlight the need for further research using robust methodologies to definitively establish the efficacy and safety of phytotherapy for managing age-related hair loss.

### Clinical studies risk of bias assessment

The following risk of bias assessment examines the methodological quality of the included clinical trials to evaluate the reliability of their findings. A comprehensive evaluation of potential biases is essential to determine the validity and strength of evidence supporting the use of phytotherapy for hair loss. The assessment was conducted using the Cochrane ROB2 tool as previously elaborated in the methodology section, supported by its crib sheet and Excel-based tool. This tool evaluates the risk of bias across several domains pertinent to study design and conduct, generally including the randomization process, blinding of participants and personnel, blinding of outcome assessment, incomplete outcome data, selective reporting, and other biases. Based on this assessment and established criteria, each trial was categorized as having a low risk of bias, some concerns, or a high risk of bias. The shown figure (Fig. 5) presents summaries of the risk of bias assessments, and the following sections provide a thorough discussion of the findings, highlighting both the methodological strengths and limitations of the included studies.

#### Low risk of bias domains

Several trials demonstrated methodological strengths and were judged to have a low risk of bias in specific domains, reflecting robust trial designs that enhance the credibility of their findings.

The studies conducted by Akhbari et al. (2024) and (Ashraf et al. 2022) showcased strong blinding protocols with triple- and double-blind designs, respectively. These blinding strategies effectively minimized the probability of performance and detection biases due to deviations from intended interventions by ensuring that participants, caregivers, and researchers remained unaware of treatment assignments throughout the study. As a result, the outcomes reported in these trials can be attributed more confidently to the interventions themselves rather than to any influence from the trial personnel or participants.

Similarly, the rigorous randomization process was a key feature of the trials conducted by Cheyasak et al. (2024); Lueangarun and Panchaprateep 2020), and (Masoud et al. 2020). These studies employed computer-generated random sequences and implemented proper allocation concealment methods, such as sealed envelopes or automated systems, ensuring that intervention allocation was unbiased and unpredictable. The meticulous application of these procedures reduced the risk of selection bias and contributed to the validity of their findings.

The trial by Mirgaloybayat et al. (2024) further enhanced its randomization method by using sealed, opaque, and sequentially numbered boxes to conceal allocation until the point of participant assignment. This added layer of accuracy safeguarded the integrity of the randomization process, ensuring that neither participants nor researchers could manipulate group assignments.

In addition to strong randomization and blinding practices, (Xu et al. 2021) demonstrated a low risk of bias in the domain of missing outcome data. The study reported complete follow-up for all 80 randomized participants, with no missing data for primary or secondary outcomes. This level of retention and complete dataset strengthen the reliability of the results, as it eliminates concerns regarding potential bias introduced by subject attrition.

#### Some concerns in risk of bias domains

A notable number of the included trials exhibited “some concerns” in specific domains. This judgment indicates that while there is no clear indication of a high risk of bias, certain aspects of the study design or reporting raise methodological uncertainties that warrant careful consideration and could influence the reliability of their findings.

One repeated issue across multiple studies was the lack of explicit reporting on allocation concealment methods. Adequate allocation concealment is crucial to ensure that researchers enrolling participants are unaware of the upcoming treatment allocation. This prevents them from consciously or unconsciously influencing the assignment process. Trials such as (Akhbari et al. 2024; Feldman et al. 2022; Kim et al. 2020; Somboonwatthanakul et al. 2024), and (Tanuphol et al. 2024) did not provide sufficient details on how allocation sequences were concealed from investigators and participants. Without clear and robust documentation of these procedures, it remains uncertain whether the assignment process was fully protected from manipulation or bias.

Another common limitation was the inadequate reporting of blinding procedures for outcome assessors. Blinding outcome assessors who are measuring the primary and secondary outcomes is essential to prevent their knowledge of the treatment allocation from influencing their assessments. Although some studies explicitly mentioned blinding, others, including (Pekmezci et al. 2018; Masoud et al. 2020; Somboonwatthanakul et al. 2024), and (Tanuphol et al. 2024), did not deliver sufficient information to confirm whether those assessing outcomes were blinded to treatment allocation. This lack of transparency raises concerns about the likelihood of detection bias, particularly for subjective outcomes such as patient-reported hair regrowth or satisfaction.

A third area of concern was the absence of pre-specified analysis plans. A pre-specified analysis plan, ideally registered before the start of the trial, outlines the primary and secondary outcomes, statistical methods, and subgroup analyses that will be conducted. Trials such as (Cheyasak et al. 2024; Pekmezci et al. 2018; Tanuphol et al. 2024; Masoud et al. 2020; Moosavi et al. 2020; Ashraf et al. 2022; Yu et al. 2019; Somboonwatthanakul et al. 2024), and (Xu et al. 2021) did not indicate whether their statistical analyses and outcome measures were defined prior to the beginning of the study. This oversight increases the risk of selective reporting bias, as researchers may have been influenced by observed results to alter the reported outcomes or statistical methods, thereby compromising the objectivity of the findings.

#### High risk of bias domains

Several trials were found to have a high risk of bias in one or more domains, demanding cautious interpretation of their findings due to significant methodological limitations.

High attrition rates were a prominent risk of bias due to missing outcome data in trials such as (Jae-Suk et al. 2015; Feldman et al. 2022), and (Kim et al. 2020), where substantial proportions of participants withdrew or were excluded from the final analysis. The absence of detailed explanations raises concerns about the potential for systematic differences between those included and excluded. Additionally, the lack of information regarding the reasons for withdrawal makes it difficult to determine whether missingness was related to the effectiveness or safety of the therapy. Such biases in missing outcome data can distort the estimated treatment effects, particularly if attrition was differential across intervention arms.

The trials conducted by Campiche et al. (2022) and (Somboonwatthanakul et al. 2024) exhibited deficiencies in blinding procedures, which introduced a high risk of both performance and detection biases due to potential deviations from intended interventions and outcome measurement. These trials did not adequately blind participants, caregivers, or outcome assessors and, in some cases, relied on subjective self-reported outcomes. This lack of methodological rigor diminishes the credibility of the findings, as knowledge of treatment allocation may have influenced both participant behaviors and outcome measurements.

Even trials with otherwise strong designs, such as (Akhbari et al. 2024), were not resistant to high risks of bias in certain domains related to missing outcome data. In this trial, a significant number of participants failed to complete the study, and the reasons for withdrawal were not sufficiently documented. Such incomplete data, coupled with the lack of information about the dropout causes, weaken the reliability of the trial’s conclusions and raise concerns about the potential impact of missing data on the results, as the missing information may obscure potential differences between treatment groups.

#### Overall risk of bias and evidence quality

The overall risk of bias across the included trials ranged from “Some Concerns” to “High” as illustrated in the following figure (Fig. 6), indicating that the evidence supporting the use of phytotherapy for hair loss varies in quality. While some trials demonstrated creditable methodological rigor, others exhibited critical limitations, such as insufficient allocation concealment, incomplete blinding, and poorly managed missing data. So, trials with a high risk of bias may overestimate the true effects of the interventions, potentially leading to misleading conclusions. These methodological flaws highlight the variability in the quality of evidence supporting the use of phytotherapy for hair loss. Consequently, it is crucial to consider the weaknesses of each study and interpret the findings cautiously.

The presence of high-risk domains in several trials suggests that the reported treatment effects may be subject to overestimation, necessitating cautious interpretation. However, the findings from trials with low risk of bias provide a stronger foundation for evaluating the efficacy and safety of phytotherapeutic interventions. This assessment highlights the importance of future research addressing the identified methodological gaps and producing more robust data for the effectiveness and safety of phytotherapy in managing hair loss. Specifically, trials should prioritize rigorous allocation concealment, comprehensive blinding strategies, pre-registration of protocols, and transparent reporting of all planned and conducted analyses. By adhering to these best practices, future research can generate more reliable evidence and contribute to a more comprehensive understanding of the potential role of phytotherapy in managing hair loss and improving patient outcomes.

### Evidence summary of findings

Many of the trials investigating the use of phytotherapy for hair loss incorporated photographic assessment to visually evaluate changes in hair growth and density. (Jae-Suk et al. 2015) utilized standardized global photographs, reviewed by an expert panel of dermatologists, to assess hair regrowth using a 7-point scale. Similarly, (Campiche et al. 2022) presented scalp microphotographs using Trichoscan to evaluate hair density. Additionally, (Lueangarun and Panchaprateep 2020) employed both global and dermoscopic photographs of a target area with a 1-cm^2^ grid to allow blinded dermatologists to count hairs per square centimeter. Besides, (Cheyasak et al. 2024) presented “before and after” photographs to illustrate hair conditions in participants of both the herbal solution and minoxidil groups. (Panahi et al. 2015) also provided sample pre-trial and post-trial scalp photographs from both the rosemary and minoxidil groups. Finally, (Tanuphol et al. 2024) included representative vertex area photographs of subjects in the minoxidil, HT-teak, and placebo groups at baseline and week 24. These photographic assessments provided visual evidence to support the quantitative data on hair growth and density, offering a more comprehensive understanding of the treatment effects. Refer to the following table of “before and after” hair pictures for a visual representation of these findings across the eligible trials (Table 5).

**Table 5:** “Before and after” hair visual representations in hair loss phytotherapy-using clinical trials

Study ID	Visualization method	Hair before and after
Campiche et al. (2022)	TrichoScan® to measure scalp hair density	**Fig. 7.** Scalp microimages showing hairs (black dots) at baseline (**A**) and after 5 months of using 3% Edelweiss CB formulation (**B**). Adapted from Campiche et al. 2022, International journal of cosmetic science, licensed under CC-BY 4.0 (https://creativecommons.org/licenses/by/4.0/).
Cheyasak et al. (2024)	Dermoscopy to measure scalp hair density and Global photography to measure hair growth	**Fig. 8.** Dermoscopic pictures showing hair density at baseline (**A**) and after using Redenyl® herbal extract for 6 months (**B**). Adapted from Cheyasak et al. 2024, Open Dermatology Journal, licensed under CC-BY -NC 4.0 (https://creativecommons.org/licenses/by/4.0/). **Fig. 9.** Global photographs showing hair growth at baseline (**A**) and after using Redenyl® herbal extract for 2 months (**B**), 4 months (**C**), and 6 months (**D**). Adapted from Cheyasak et al. 2024, Open Dermatology Journal, licensed under CC-BY -NC 4.0 (https://creativecommons.org/licenses/by-nc/4.0/).
Choi et al. (2015)	Global Photography to measure hair growth	**Fig. 10.** Global photographs showing hair growth at baseline (**A**) and after using Rice bran SC extract for 2 weeks (**B**) and 1 month (**C**). Reproduced with permission from Choi et al. 2015, Biological & Pharmaceutical Bulletin. Copyright © Pharmaceutical Society of Japan.
Lueangarun and Panchaprateep (2020)	Global photography to measure clinical improvement in hair loss and dermoscopy with 1-cm² grid to measure hair count	**Fig. 11.** Global vertex photographs showing male pattern hair loss at baseline (**A**) and after using Hirsuit™ herbal tonic for 3 months (**B**) and for 6 months (**C**). Reproduced with permission from Lueangarun & Panchaprateep, 2020, The Journal of clinical and aesthetic dermatology. Copyright © Journal of Clinical and Aesthetic Dermatology. **Fig. 12.** Global vertex photographs showing female pattern hair loss at baseline (**A**) and after using Hirsuit™ herbal tonic for 3 months (**B**) and for 6 months (**C**). Reproduced with permission from Lueangarun & Panchaprateep, 2020, The Journal of clinical and aesthetic dermatology. Copyright © Journal of Clinical and Aesthetic Dermatology. **Fig. 13.** Dermoscopic pictures showing male pattern hair loss (**A**) and female pattern hair loss (**C**) at baseline and after using Hirsuit™ herbal tonic (**B**) and (**D**) respectively. Reproduced with permission from Lueangarun & Panchaprateep, 2020, The Journal of clinical and aesthetic dermatology. Copyright © Matrix Medical Communications.
Panahi et al. (2015)	Microphotographic assessment to measure hair count	**Fig. 14.** Scalp photographs showing hair count at baseline (**A**) and after using Rosemary oil for 6 months (**B**). Reproduced with permission from Panahi et al. 2015, Skinmed. Copyright © Pulse Marketing and Communications, LLC.
Tanuphol et al. (2024)	Trichoscopy (Leviacam®) to measure hair density and count, and visual examination to measure hair regrowth	**Fig. 15.** Trichoscan pictures showing scalp target area hair count at baseline (**A**) and after using 1% teak leaf extract for 6 months (**B**); Color code = Hair type: green=anagen, red=telogen, blue=terminal, yellow=vellus. Adapted from Tanuphol et al. 2024, Journal of evidence-based integrative medicine, licensed under CC-BY -NC 4.0 (https://creativecommons.org/licenses/by-nc/4.0/). **Fig. 16.** Vertex area photographs showing hair growth at baseline (**A**) and after using 1% teak leaf extract for 6 months (**B**). Adapted from Tanuphol et al. 2024, Journal of evidence-based integrative medicine, licensed under CC-BY -NC 4.0 (https://creativecommons.org/licenses/by-nc/4.0/).

The accumulating evidence from various clinical trials suggests that phytotherapy holds significant promise as a safe and potentially effective approach to managing hair loss of diverse etiologies. Trials have revealed positive results with a range of plant-based extracts, showing improvements in hair count, hair density, anagen/telogen ratio, and overall hair growth, as demonstrated in the presented summary of findings table (Table 6). Notably, some trials have reported comparable efficacy between phytotherapeutic interventions and conventional treatments like minoxidil, further reinforcing the prospect of herbal remedies in this field. The aforementioned visual evidence from photographic assessments, utilizing methods such as Folliscope®, Trichoscan®, and global photography, provide compelling support for the observed improvements. However, it is crucial to acknowledge that more rigorous research is needed to solidify these findings and translate them into widely accepted clinical practices. Future research should prioritize larger, multi-center trials with longer follow-up periods to confirm the long-term efficacy and safety of phytotherapy for hair loss. Standardization of plant extracts should also be considered to ensure consistent dosing and quality control across studies. Nevertheless, head-to-head comparisons between phytotherapeutic interventions and standard treatments should be studied to establish their relative efficacy and cost-effectiveness. Furthermore, the specific mechanisms of action of various plant extracts and their bioactive compounds need investigations to optimize treatment strategies. By addressing these research gaps, the field of phytotherapy can move towards establishing evidence-based guidelines and developing innovative, plant-based solutions for individuals struggling with hair loss.

**Table 6:** Summary of findings of hair loss phytotherapy-using clinical trials

Study ID	Hair loss cause	Final participants per arm	Duration	Intervention	Comparator/control	Outcome domain	Outcome measures	Conclusion
Akhbari et al. 2024	Genetic; FAGA	27 (amla group); 25 (placebo)	12 weeks	10 cc *Amla* syrup three times daily	10 cc of placebo syrup three times daily	Hair growth parameters	A/T; hair count; hair thickness (TrichoScan)	Amla syrup increased the anagen-to-telogen hair ratio and improved satisfaction compared to placebo. Amla is suggested as a safe oral treatment for FAGA, but further research is needed
						Physician satisfaction	CGI-I questionnaire	
						Patient satisfaction	PGI-I questionnaire	
Ashraf et al. 2022	Hormonal; hypothyroidism	27 (ginger group); 26 (placebo group)	30 days	500 mg ginger powder capsules twice daily (30 min before lunch and dinner)	500 mg starch-containing placebo capsules twice daily (30 min before lunch and dinner)	Hair loss severity (among hypothyroid symptoms)	ThySRQ	Daily consumption of 1000 mg of ginger powder for 30 days resulted in a significant reduction in hypothyroid symptoms and improvements in anthropometric measures and laboratory indices in patients with primary hypothyroidism. Although ginger was shown to alleviate various symptoms like weight gain, cold intolerance, and constipation, it did not have a statistically significant effect on hair loss in participants
Campiche et al. 2022	Aging; normal hair loss	In vivo: 30 (Edelweiss); 30 (placebo)	In vivo: 5 months (150 days)	In vivo: Leave-on serum containing 3% of a product containing 2% Edelweiss dry extract, applied daily at night	In vivo: Base formulation without Edelweiss extract (placebo)	In vivo: Hair density	TrichoScan®	The Edelweiss extract prolonged the anagen phase of hair follicles ex vivo and significantly increased hair density in vivo. The in vivo study also showed a strong trend toward an increased anagen-to-catagen/telogen ratio
						In vivo: Anagen-to-catagen/telogen ratio	Trichogram analysis	
Cheyasak et al. 2024	Genetic; AGA	30 (herbal extract); 30 (minoxidil)	6 months (24 weeks)	1 mL of the herbal extract (Redenyl®) applied twice daily	1 mL of 3% minoxidil solution applied twice daily	Hair growth; hair density	Total hair count (Dermoscopy), HMI (Haircheck system®); Dermatologist assessment (Global photography)	The herbal extract combination showed comparable efficacy and safety to minoxidil in improving hair count, suggesting it as a potential alternative treatment for AGA
						Safety/Adverse events	Reporting of adverse events	
Choi et al. 2015	Genetic; AGA	21 (RB-SCE; 9 men, 12 women); 22 (placebo; 13 men, 9 women)	16 weeks	0.5% RB-SCE applied to the scalp (total 8 mL/day as 4 mL every 12 h)	Dermal application of a placebo tonic contained no RB-SCE	Hair growth; Hair density	Hair count (Folliscope), Hair diameter (Folliscope); Dermatologist evaluation (Global photographs)	RB-SCE significantly increased hair density and hair diameter in men, with promising results in patient satisfaction and photographic evaluations and without reported adverse events, confirming its effectiveness and suggesting its potential as a treatment for androgenic alopecia
						Patient satisfaction	Self-assessment questionnaire (3-point rating scale)	
						Safety/adverse events	Subject reports; dermatologist observation	
Feldman et al. 2022	Genetic; AGA or stress; TE	37 (ALRV5XR; 20 women, 17 men); 40 (placebo; 21 women, 19 men)	24 weeks	ALRV5XR administered as oral capsule (842 mg twice daily) + daily application of shampoo (3–7 mL daily), conditioner (3–7 mL daily), and follicle serum (1 mL daily)	Matching placebo formulations for oral and topical applications	Hair density	Changes in TH and VH density (phototrichogram); TH/VH ratio	ALRV5XR showed significant improvements in terminal hair density for both men and women with AGA and TE by reactivating dormant hair follicles and thickening miniaturized (vellus-like) hairs
						Hair thickness	Hair diameter	
						Hair growth	Growth rate (hair length/day)	
						Hair shedding	Shedding rate (number of hairs shed/day)	
						Response rate	Clinical significance of TH density increase (% of participants)	
Lueangarun and Panchaprateep 2020	Genetic; AGA	17 (herbal); 15 (3% minoxidil)	24 weeks	1 mL of Hirsuit™ herbal hair tonic applied twice daily to the vertex area of the scalp	1 mL of 3% minoxidil solution applied twice daily to the vertex area of the scalp	Hair growth	Panel photographic assessment (Global scalp photographs); TAHC (Terminal hair dermoscopic photographs); HMI (HairCheck®)	The herbal extract combination showed comparable efficacy to 3% minoxidil solution for the treatment of mild-to-moderate AGA in both male and female participants in increasing terminal hair count and hair mass index. Furthermore, the herbal extract combination exhibited a favorable safety profile with no reported local adverse reactions, unlike minoxidil, suggesting it as a possible alternative treatment
						Patient satisfaction	Questionnaire (7-point rating scale)	
						Safety/toleraility	Adverse events reporting (4-point rating scale)	
Masoud et al. 2020	Genetic; AGA	12 (MTS: 5% minoxidil topical solution); 12 (MTS + THS: 5% minoxidil topical solution plus topical herbal solution)	36 weeks (9 months)	1 ml of 5% minoxidil topical solution applied in the morning and 1 ml of the topical herbal solution applied in the evening	1 ml of 5% minoxidil topical solution applied twice daily (morning and evening)	Hair growth	Hair diameter (digital micrometer); Hair density	The topical herbal solution had a significant positive effect on patients with AGA and improved their quality of life. The combination was superior to 5% minoxidil alone in improving hair diameter and increasing hair density
						Safety	Adverse events (abnormal lab tests)	
						Patient self-evaluation	Self-assessment questionnaire (quality of therapy point-scale)	
Mirgaloybayat et al. 2024	Hormonal; PCOS	55 (Fenugreek); 55 (Metformin)	8 weeks (60 days)	333 mg fenugreek capsules three times a day	Metformin 500 mg three times a day	Hair loss	Not specified	Fenugreek showed superior safety to metformin but cannot completely substitute it in treating women with PCOS. It can be considered as an adjuvant treatment in PCOS, especially in patients with hyperlipidemia and moderate to severe hair loss
Moosavi et al. 2020	Immunological; AA	22 (Squill); 20 (Clobetasol)	12 weeks	Topical Squill lotion twice daily (morning and night) rubbed on the alopecia patches	Topical Clobetasol propionate 0.05% lotion twice daily (morning and night) rubbed on the alopecia patches	Hair growth	Patches size (standardized template); hair grown no. (lens); regrowth score (semi-quantitative scale)	Squill extract demonstrated comparable efficacy to clobetasol propionate in treating AA. Its 2% lotion showed good effect in 45% patients with patchy alopecia areata and moderate effect on regrowth of terminal hairs
						Patient satisfaction	Patient’s personal opinion	
Panahi et al. 2015	Genetic; AGA	50 (rosemary oil); 50 (2% minoxidil)	6 months (24 weeks)	Topical application of rosemary essential oil twice daily to the affected areas of the scalp	Topical application of 2% minoxidil solution twice daily to the affected areas of the scalp	Hair growth	Hair count (phototrichogram)	Rosemary oil, applied topically, appears to be as effective as 2% minoxidil in the treatment of AGA. Rosemary oil was associated with a lower incidence of scalp itching as a side effect compared to minoxidil
						Scalp health	Frequency of scalp itching, dry hair, greasy hair, and dandruff	
						Participant-reported outcomes	Self-reported improvement (questionnaire)	
						Safety	Adverse events	
Pekmezci et al. 2018	Genetic; AGA or Stress; TE	30 subjects per group; 4 groups (A: herbal shampoo; B: herbal solution; C: herbal shampoo and solution; D: placebo shampoo and solution)	6 months	5 ml of Herbal extracts shampoo applied to wet hair three times a week and left to foam for 3–4 min before rinsing; 3 ml of same herbal extract solution applied to dry hair twice daily, massaged into the scalp and left for at least 4–6 h	Placebo shampoo and solution formulated to match the herbal versions in appearance and texture but without the herbal extracts	Hair loss	Pull test (Number of hairs extracted during gentle traction)	The herbal hair care product demonstrated significant improvements in hair parameters compared to baseline for both AGA and TE. No serious adverse events were reported. The combination of shampoo and solution demonstrated a synergistic effect, concluding that these products could be safe and effective treatment for hair loss
						Hair growth	Hair count (Phototrichogram); A/T (% of hair in each phase)	
						Scalp health	Clinical assessment of scalp conditions by a dermatologist	
						Self-perceived improvement	Self-assessment questionnaire (10-point ratings on hair properties)	
Somboonwatthanakul et al. 2024	Aging; Normal Hair Loss	10 (C: control tonic vs. R: experimental tonic); 10 (G: commercial tonic vs. R: experimental tonic)	30 days	R (Experimental): Topical hair tonic containing riceberry oil and other extracts*;* 5 drops (0.13 g) applied to the designated side of the scalp daily	C (Control): Hair tonic without rice by-products or herbal extracts; G (Commercial): Giffarine Herbita hair tonic; 5 drops (0.13 g) of each hair tonic applied to the designated side of the scalp daily	Hair loss	Hair count (% reduction)	The experimental hair tonic (R) effectively reduced hair loss and showed potential in reducing gray hair and dandruff comparable to the commercial hair tonic (G). It was well-received by users, highlighting its potential efficacy and safety as a natural hair care solution
						User satisfaction	A 9-point Likert scale survey	
Tanuphol et al. 2024	Genetic; AGA	27 (teak leaf); 27 (5% minoxidil); 27 (placebo)	24 weeks	Topical application of 1% teak leaf extract hair tonic twice daily (morning and night); 3–5 drops (0.5 ml) to the scalp vertex and temporal areas	Positive control: 3–5 drops (0.5 ml) of the 5% minoxidil applied twice daily; placebo: 3–5 drops (0.5 ml) of the base formulation of the HT-teak applied twice daily	Hair growth	TAHC (Leviacam®); A/T (Leviacam®)	The hair tonic with teak leaf extract demonstrated significant improvement in hair count and density, comparable to 5% minoxidil, and was well-tolerated, suggesting its potential as a natural safe and effective alternative hair growth promoter alternative for AGA treatment
						Hair shedding	Combing test	
						Adverse events	Dermatological lesions; systemic adverse events (male reproductive system issues and palpitations)	
						Satisfaction	Self-reported score (7-point scale)	
Xu et al. 2021	Medications; CIA	40 (TCM + chemotherapy); 40 (chemotherapy alone)	6 months	A trastuzumab plus paclitaxel chemotherapy regimen and personalized TCM taken twice a day	A trastuzumab plus paclitaxel chemotherapy regimen alone	Hair loss (among medication-related side effects)	Hair loss severity	TCM combined with chemotherapy effectively reduced the severity of chemotherapy-induced hair loss and improved quality of life compared to chemotherapy alone. It also mitigated other common side effects, supporting its use as an adjunctive therapy in breast cancer patients
Yu et al. 2019	Medications; CIA	47 (routine chemotherapy only); 46 (Xiaoaiping + chemotherapy)	Not specified (from January 2016 to December 2017)	Routine chemotherapy regimen plus Xiaoaiping (7.2 g/day, three times daily) for two weeks during each chemotherapy cycle	Six chemotherapy cycles of docetaxel (75 mg/m^2^) and adriamycin (50 mg/m^2^) every 21 days alone	Alopecia	Alopecia severity (WHO criteria)	Xiaoaiping combined with chemotherapy was found to be more effective than chemotherapy alone in reducing hair loss severity and improving quality of life in breast cancer patients. It also demonstrated a positive impact in mitigating other side effects, suggesting its potential as a supportive treatment
							Alopecia-free time (Grade scale)	
						Quality of life	FACT-B score	

*A/T* anagen-to-telogen ratio, *AA* alopecia areata, *AGA* androgenetic alopecia, *CGI-I* Clinical Global Impression of–Improvement scale, *CIA* chemotherapy-induced alopecia, *FAGA* female androgenetic alopecia, *HMI* Hair Mass Index, *PCOS* polycystic ovary syndrome, *PGI-I* Patient Global Impression of Improvement, *RB-SCE* rice bran supercritical CO_2_ extract, *TAHC* target area hair count, *TCM* traditional Chinese medicine, *TE* telogen effluvium, *TH* terminal hair, *ThySRQ* Thyroid Symptom Rating Questionnaire, *VH* vellus hair

## Conclusions

This literature indicates that herbal drugs have promising efficacy in hair loss control, and many of them show potential activity in the treatment with multiple mechanisms. For example, such activity includes promoting vasculature and follicular functions, showing hormonal regulation, and having anti-inflammatory properties. Despite these encouraging findings, significant challenges remain while interpreting them due to several limitations in the available clinical evidence. Important caveats are small sample sizes, heterogeneity in terms of study designs, and missing standardization of protocols. Furthermore, the absence of standardized herbal preparations and processing, along with differences in dosage, formulation, application methods, and intake techniques, hinders comparisons in results. Although there is strong preclinical evidence for several herbs, evidence of their translational potential in clinical settings is often weak because of methodological discrepancies. Future work is warranted in high-quality, large-scale, randomized controlled trials employing standardized herbal interventions to substantiate and more reliably assess the therapeutic benefit of herbal medicine. Additionally, exploring synergistic effects between herbal and conventional treatments may offer new avenues for comprehensive hair loss management. Overall, this review emphasized the importance of rigorous systematic evaluation and highlights the critical role of evidence-based approaches when considering incorporating herbal medicine into standard dermatological practice.

## Supplementary Information

Below is the link to the electronic supplementary material.

Supplementary file1 (XLSX 25 KB)

## Data Availability

All source data for this work (or generated in this study) are available upon reasonable request.
